# Characterization of histone deacetylases and their roles in response to abiotic and PAMPs stresses in *Sorghum bicolor*

**DOI:** 10.1186/s12864-021-08229-2

**Published:** 2022-01-06

**Authors:** Qiaoli Du, Yuanpeng Fang, Junmei Jiang, Meiqing Chen, Xiaodong Fu, Zaifu Yang, Liting Luo, Qijiao Wu, Qian Yang, Lujie Wang, Zhiguang Qu, Xiangyang Li, Xin Xie

**Affiliations:** 1 Key Laboratory of Agricultural Microbiology, College of Agriculture, Guizhou University, Guiyang, 550025 PR China; 2 State Key Laboratory Breeding Base of Green Pesticide and Agricultural Bioengineering, Key Laboratory of Green Pesticide and Agricultural Bioengineering, Ministry of Education, Guizhou University, Guiyang, 550025 PR China

**Keywords:** Genome analysis, Histone deacetylases, Expression profile, Abiotic stress, Pathogen-associated molecular model (PAMPs), *Sorghum bicolor*

## Abstract

**Background:**

Histone deacetylases (HDACs) play an important role in the regulation of gene expression, which is indispensable in plant growth, development, and responses to environmental stresses. In *Arabidopsis* and rice, the molecular functions of HDACs have been well-described. However, systematic analysis of the *HDAC* gene family and gene expression in response to biotic and abiotic stresses has not been reported for sorghum.

**Results:**

We conducted a systematic analysis of the sorghum *HDAC* gene family and identified 19 *SbHDACs* mainly distributed on eight chromosomes. Phylogenetic tree analysis of SbHDACs showed that the gene family was divided into three subfamilies: RPD3/HDA1, SIR2, and HD2. Tissue-specific expression results showed that *SbHDACs* displayed different expression patterns in different tissues, indicating that these genes may perform different functions in growth and development. The expression pattern of *SbHDACs* under different stresses (high and low temperature, drought, osmotic and salt) and pathogen-associated molecular model (PAMPs) elf18, chitin, and flg22) indicated that *SbHDAC* genes may participate in adversity responses and biological stress defenses. Overexpression of *SbHDA1*, *SbHDA3*, *SbHDT2* and *SbSRT2* in *Escherichia coli* promoted the growth of recombinant cells under abiotic stress. Interestingly, we also showed that the sorghum acetylation level was enhanced when plants were under cold, heat, drought, osmotic and salt stresses. The findings will help us to understand the *HDAC* gene family in sorghum, and illuminate the molecular mechanism of the responses to abiotic and biotic stresses.

**Conclusion:**

We have identified and classified 19 *HDAC* genes in sorghum. Our data provides insights into the evolution of the *HDAC* gene family and further support the hypothesis that these genes are important for the plant responses to abiotic and biotic stresses.

**Supplementary Information:**

The online version contains supplementary material available at 10.1186/s12864-021-08229-2.

## Background

Epigenetic regulation is an important regulatory mechanism that helps plants adapt to environmental stresses. Moreover, epigenetic regulation of gene expression proceeds through DNA or histone modification without changing the DNA sequence, which is fast, reversible, and heritable [1]. Chromatin remodeling, an important type of epigenetic regulation, is one of the key regulators of gene expression in higher plants. It affects various cellular processes by regulating gene expression in different growth and development stages [2]. Reports have shown that different environmental stresses can cause different types of modifications in histones [3]. For example, histone post-translational modifications mainly include histone acetylation, ubiquitination, phosphorylation, ADP-ribosylation, and methylation [4]. Among these, histone acetylation has attracted the most attention, and histone acetylation modification plays a vital role in the regulation of eukaryotic transcription activity. Histone acetylation mainly occurs on lysine residues at histone tails. It is a reversible dynamic equilibrium process, mainly controlled by histone acetyltransferases (HATs) that catalyze histone acetylation and histone deacetylases (HDACs) that control the co-regulation of histone deacetylation. HATs transfer the acetyl group of the acetyl-CoA to lysine residues at the end of histones to eliminate the positive charge and force the chromatin structure into a more elongated state, which is beneficial for transcription factor binding and is related to transcriptional activation of genes. Meanwhile, HDACs remove the acetyl groups from the ends of histones, leaving chromatin in a tighter, more condensed state, which is not conducive to the binding of transcription factors or transcriptional regulators to DNA, and is associated with transcriptional suppression/silencing [5]. Thus, histone modification plays an important role in the regulation of gene expression; HATs promote gene expression, and HDACs inhibit gene expression.

Histone acetylases (HATs) are divided into five families based on sequence characteristics: GNAT, MYST, p300/CREB binding protein (CBP) coactivator, TAFII250, and HATs. HDACs in plants are divided into three subfamilies: RPD3/HDA1, SIR2, and HD2 [6], the first two of which are homologous to the yeast RPD3/HDA1 and SIR2 families, and RPD3/HDA1 is the largest subfamily of the *HDAC* family. These HDACs are mainly distributed in the nucleus or cytoplasm, or they shuttle between the nucleus and cytoplasm [7]. Members of the SIR2 family are conserved from prokaryotes to eukaryotes, and use Nicotinamide adenine dinucleotide (NAD^+^) as a coenzyme to regulate the activity of *HDACs* [8]. The HD2 family is unique to plants and has not been found in yeasts or animals [9, 10]. In *Arabidopsis*, HD2 family members have a conserved amino acid region (EFWG motif) at the N-terminus, and both HDT1 and HDT3 contain a C2H2 zinc finger domain that may mediate DNA-protein or protein-protein interactions [11]. All members of this family contain a typical histone deacetylase domain, the enzymatic activity of which requires the presence of Zn^2+^. A large number of studies have shown that in the human body, *HDACs* are closely related to the occurrence and development of cancer [12]. In 1988, *HDACs* were also found in plants [13]. Histone acetylation and deacetylation play an important role in the growth and development of plants, including root development [14], flower development [15], gametophyte development [16], and cell proliferation during organ growth [17]. They also participate in plant responses to changes in the external environment, such as light signals [18], salt stress and abscisic acid (ABA) signaling [19], cold stress [20], heat stress [21] and other hormone signaling pathways [22].

Members of the *HDAC* gene family have been identified in *Arabidopsis* [23], rice [24], corn [9], tomato [25], cotton [26], tea [27], and other plants. Eighteen *HDAC* members have been identified in the *Arabidopsis* genome, all of which belong to the above three *HDAC* families. Increasing evidence shows that in the response of *Arabidopsis* to biological and abiotic stresses, *HDACs* play a vital role in the process of epigenetic regulation. For example, *AtHDA6* and *AtHDA19* function in the abscisic acid (ABA) signaling pathway, and can also be induced by jasmonic acid (JA) and ethylene, while HDA6 interacts with EIN3 and HDA proteins in ethylene and JA signaling [28]. *AtHDA19* inhibits WRKY38 and WRKY62 transcription factors and regulates the expression of Pathogenesis Related 1 (PR1) by participating in the plant defense response mediated by salicylic acid (SA) [29]. Overexpression of *AtHD2C* in *Arabidopsis* leads to increased tolerance to salt and drought stress [30]. HD2 family members also play a cardinal role in plant growth and development. For example, silencing the *HD2A* gene in *Arabidopsis* causes seed development to cease [31], while overexpression of *HD2A* alters leaf and flower morphology [11], defects, delayed flowering, and suspension of seed development. Expression of *HD2A*, *HD2B*, *HD2C*, and *HD2D* is inhibited by ABA and sodium chloride [19]. In rice, *HDT705* participates in the regulation of seed germination in response to abiotic stress [32]. Research on *HDAC* genes in rice showed that overexpression of *HDT701* in transgenic rice leads to increased susceptibility to rice blast (*Magnaporthe oryzae*) and bacterial blight (*Xanthomonas oryzae* pv *oryzae* (Xoo), but silencing of the *HDT701* gene increases resistance to these two pathogens, and stimulates the production of reactive oxygen species (ROS) under the action of the PAMP elicitors flg22 and chitin. The level of histone H4 acetylation has a clear regulatory effect; it negatively regulates the transcription of defense-related genes, indicating that *HDT701* regulates the level of histone H4 acetylation of pattern recognition receptors and defense-related genes [33]. SIR2 proteins are also important in the response to pathogen infection. For example, the SIR2 family protein AtSRT2 in *Arabidopsis* plays a negative regulatory role in the basic defense of plants against the pathogen PstDC3000, and expression of the *AtSRT2* gene is inhibited under pathogen infection [34]. In rice, the *OsSRT1* gene inhibits the expression of starch metabolism-related genes in seeds [35]. Reports showed that different *HDAC* family members have been found to mediate different aspects of plant growth and development. They also play a role in biological and abiotic stress responses, but the characteristics of *HDAC* genes in sorghum have not yet been reported.

In the present study, we identified 19 *HDAC*-encoding genes in sorghum. Using bioinformatics, we systematically analyzed phylogenetic relationships, conserved domains, and motifs of the sorghum *HDAC* gene family, and we performed real-time fluorescence quantitative PCR (qRT-PCR) to assess the responses of the 19 genes in different tissues and under different stresses. Changes in acetylation in sorghum seedlings were assessed under adverse stress conditions (low and high temperature, NaCl, PEG 6000). In addition, we also performed prokaryotic expression analysis of *SbHDA1*, *SbHDA3*, *SbHDT3*, and *SbSRT2* genes, and carried out spot experiments under heat, salt, osmotic and drought stresses, to verify whether expression of these genes in *E. coli* could enhance stress tolerance. The findings lay a molecular foundation for further exploring the functions of sorghum *HDAC* genes in sorghum resistance breeding.

## Results

### Genome-wide identification of *HDAC* genes in *Sorghum bicolor*

We performed a comprehensive identification of *HDAC* genes in sorghum genome, and removed the redundant *HDAC* genes based on conserved domains. Totally, 19 HDAC protein sequences containing conserved histone deacetylase domains were obtained. Bioinformatics analysis showed that genes encoded proteins ranging from 269 (SbHDT1) to 703 (SbHDA2) amino acid residues. The coding sequences of the sorghum *SbHDAC* gene family range from 834 bp (*SbHDT2*) to 1905 bp (*SbHDA1*), the molecular weight of the corresponding proteins ranges from 29.54679 (SbHDT1) to 45.30 (SbHDA2) kDa, and the pI of most SbHDACs proteins is < 7.0, making them acidic at neutral pH (Table 1), except for SbHDT2 (pI = 7.92), SbSRT1 (pI = 8.88), and SbSRT2 (pI = 9.17). The 19 *HDAC* genes are distributed on nine chromosomes; chromosome 3 contains the largest number of *HDAC* genes (4), followed by chromosomes 9 and 10 (3), chromosomes 2, 4, and 6 each contain 2, chromosomes 5, 7, and 8 each contain 1, and chromosome 1 does not contain any *HDAC* genes (Fig. S1).

**Table 1:** Identification of *SbHDAC* gene family members

Gene Name	Accession Number	Chr	Range	CDS (bp)	Protein (aa)	Molecular weight (kDa)	PI	Subcellular localization
*SbHDA1*	LOC110432512	2	4,103,729 ~ 4,115,614	1905	634	68.29599	5.68	Nucleus
*SbHDA2*	LOC8060653	2	72,626,015 ~ 72,634,000	2112	703	76.72727	5.78	Chloroplast. Nucleus.
*SbHDA3*	LOC8079300	3	52,802,297 ~ 52,806,221	1404	467	51.90450	5.78	Nucleus
*SbHDA4*	LOC8085432	3	66,687,911 ~ 66,692,549	1203	430	46.69788	6.13	Nucleus
*SbHDA5*	LOC8082952	4	7,759,256 ~ 7,763,600	1551	516	57.75734	5.29	Nucleus
*SbHDA6*	LOC8085534	4	8,149,916 ~ 8,155,266	1059	352	38.76319	5.51	Chloroplast. Nucleus.
*SbHDA7*	LOC8056459	5	10,412,638 ~ 10,414,364	1077	358	38.86902	9.08	Nucleus
*SbHDA8*	LOC8075265	6	42,785,502 ~ 42,802,511	1344	447	50.82971	6.55	Cytoplasm. Nucleus
*SbHDA9*	LOC8073075	7	47,337,650 ~ 47,352,155	1377	458	50.95214	5.43	Cytoplasm. Nucleus.
*SbHDA10*	LOC8058527	9	50,989,573 ~ 50,993,387	1173	390	42.25157	5.49	Nucleus
*SbHDA11*	LOC8079300	10	50,823,203 ~ 50,827,657	1053	350	38.61221	6.01	Chloroplast. Nucleus.
*SbHDA12*	LOC8065038	10	51,422,040 ~ 51,430,431	1557	518	57.76654	5.48	Cytoplasm. Nucleus.
*SbHDT1*	LOC8078709	3	70,646,814 ~ 70,650,720	810	269	29.54679	5.56	Nucleus
*SbHDT2*	LOC8078710	3	70,659,296 ~ 70,662,320	834	277	29.70739	5.02	Nucleus
*SbHDT3*	LOC8068982	9	55,768,231 ~ 55,771,439	900	299	32.30415	4.64	Nucleus
*SbHDT4*	LOC8066575	9	59,367,712 ~ 59,370,487	924	307	33.37051	4.80	Nucleus
*SbHDT5*	LOC8065099	10	58,010,970 ~ 58,014,442	1461	486	52.29158	7.92	Nucleus
*SbSRT1*	LOC110436344	6	48,919,950 ~ 48,935,447	1431	476	53.23976	8.88	Chloroplast
*SbSRT2*	LOC8076304	8	6,041,680 ~ 6,047,145	1455	484	53.66489	9.17	Chloroplast

E-value≤1 × 10^−5^ was used to identify the *HDAC* gene family members in sorghum as the minimum threshold

### Phylogenetic analysis and sequence alignment of SbHDACs

**Fig. 1:**
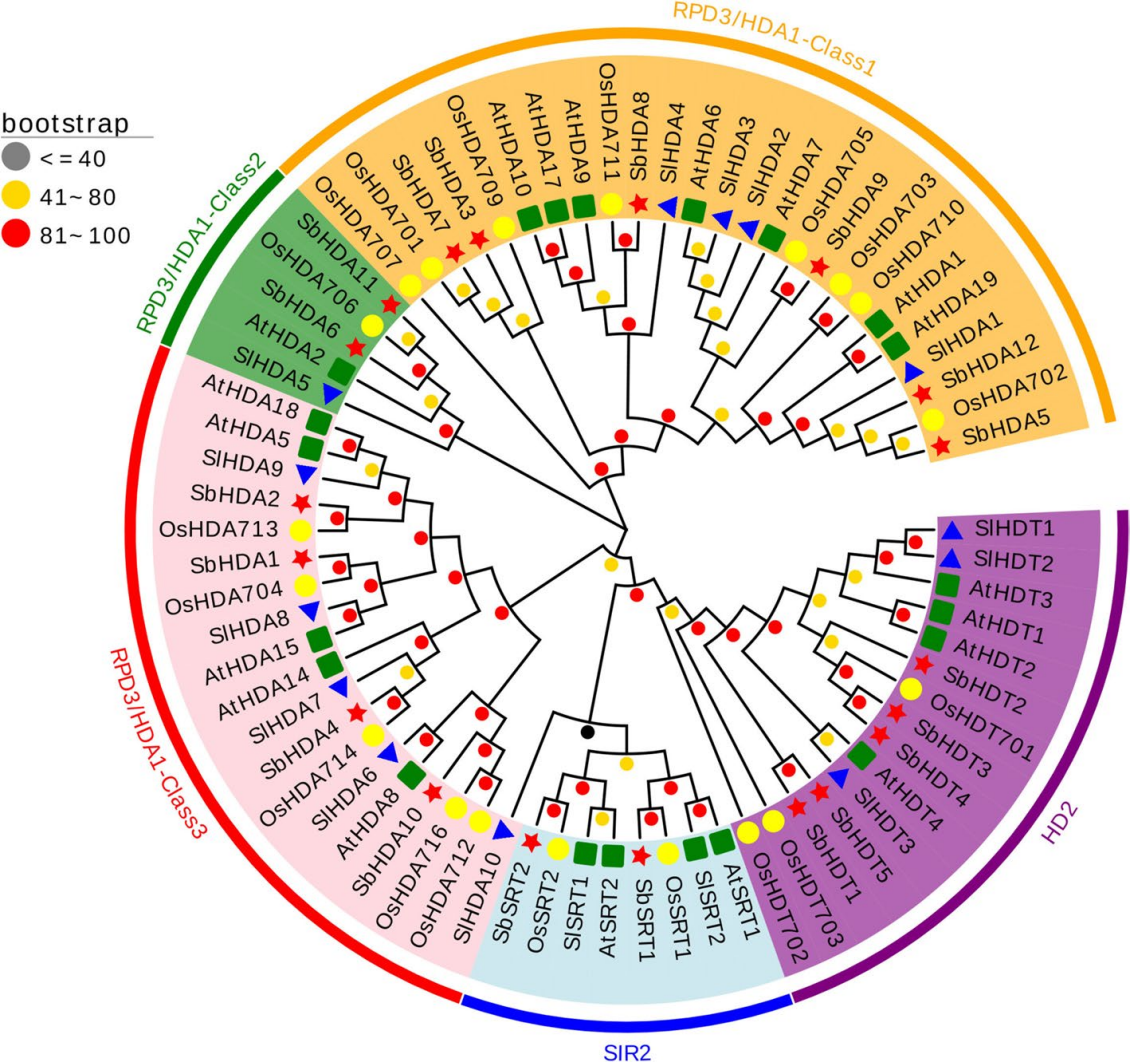
Phylogenetic relationship of *HDAC* gene family among sorghum, tomato, rice, and *Arabidopsis*. Multiple sequences alignment and phylogenetic tree were constructed by MEGA7.0 and the bootstrap test was performed with 1000 iterations. The five groups are indicated with camber lines

Using the amino acid sequences of *HDACs* in *Arabidopsis*, rice, and tomato, together with *HDACs* in sorghum, MEGA7.0 was employed to construct a phylogenetic tree of the *HDAC* gene family (Fig. 1). The HDAC sequences of sorghum are clustered into three subfamilies: RPD3/HDA1, SIR2, and HD2. Among them, RPD3/HDA1 has the most member (12). Based on sequence similarity, this subfamily can be divided into RPD3/HDA1-Class1, RPD3/HDA1-Class2, and RPD3/HDA1-Class3, each group contains 6, 2 and 4 sorghum SbHDAC members., respectively. All members of the RPD3/HDA1 subfamily share similarity with the HDAC domain of sorghum; only two SIR2 members containing the SIR2 domain are found in sorghum. In addition, sorghum HD2 contains five members with a C2H2 zinc finger domain that recognize and bind DNA, indicating that they may bind DNA or mediate protein-protein interactions. DNAMAN7.0 software was used to compare the amino acid sequences of members of the sorghum SbHDAC family. The results showed that the overall sequence identity of the 19 SbHDAC proteins was only 16.59% (Fig. S2).

### Collinearity analysis of the *SbHDACs* gene family

**Fig. 2:**
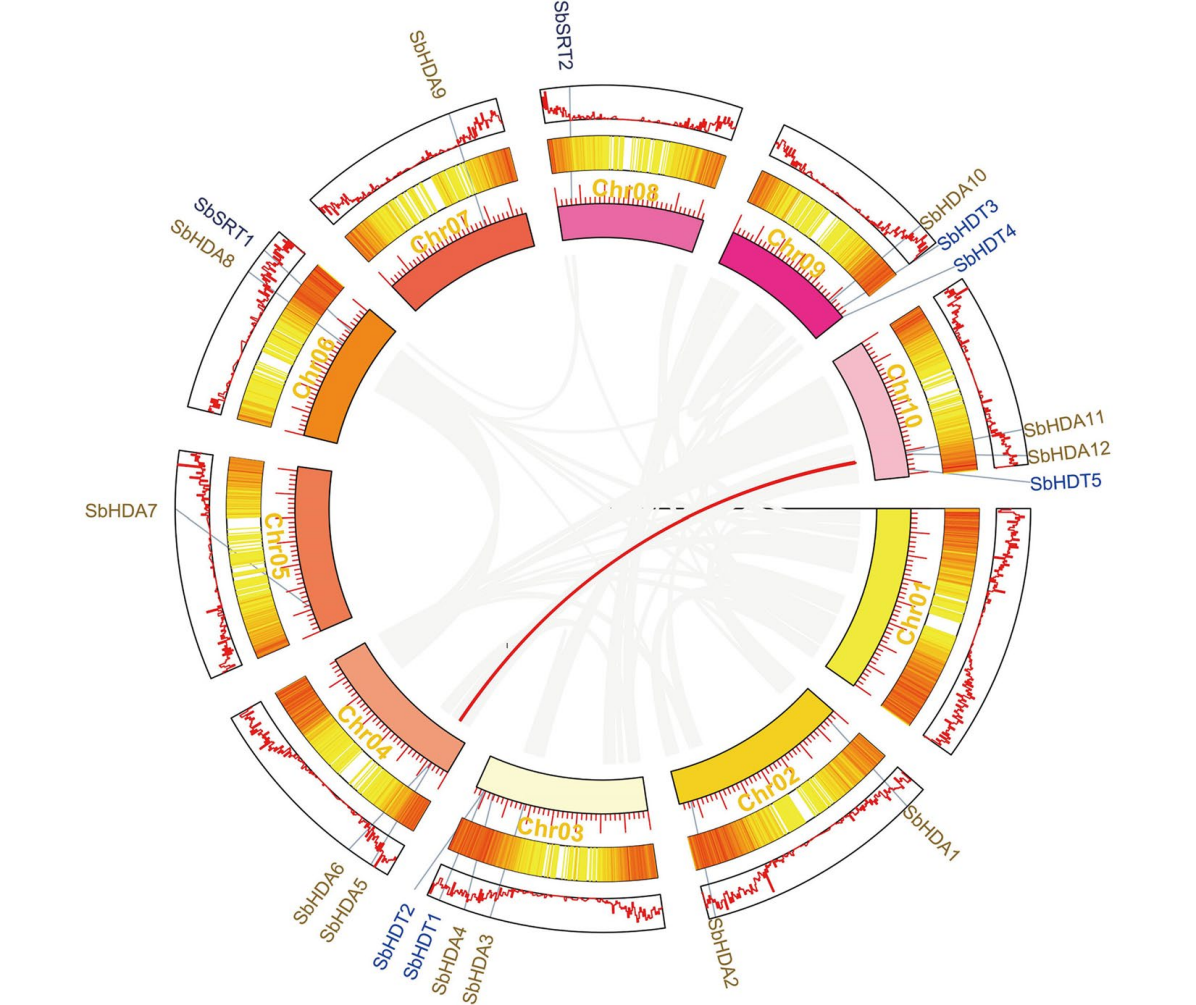
Distribution of collinearity gene pairs of *SbHDAC* genes in sorghum. The red line indicates that a pair of collinearity gene (*SbHDA5*/*SbHDA11*). Collinearity is caculated by the MCScanX verson1.0 software

Collinearity analysis of the sorghum *HDAC* gene family identified only one pair of collinearity gene (*SbHDA5*/*SbHDA11*) pair in sorghum genome, indicating a replication event may have occurred in the recent past. Besides, this event did not cause significant amplification of HDACs, or significant gene loss after genome duplication event (Fig. 2).

### Gene structure and conserved motifs of SbHDACs proteins

In order to further explore the structural characteristics of the sorghum *HDAC* gene family, we analyzed intron-exon numbers and conserved motifs of all members. The results showed that the number of introns and exons in *SbHDAC* genes was 0-15 and 1-18, respectively (Fig. S3). And there are almost no differences between members of the HD2 subfamily. The more closely related genes in the phylogenetic tree appeared to have similar structural components, hence it can be concluded that genes in the same subgroup may perform similar functions.

In order to further analyze the diversity of sorghum SbHDACs, 30 conserved motifs were predicted using MEME online software. In general, proteins clustered in the same subfamily share similar motifs, indicating that family protein members in the same subfamily may have similar functions. As can be seen in Fig. S4, all 12 members of the HDA contain motifs 1 and 5; the five members of the HD2 subfamily all contain motif 7, motif 9, motif 16, and motif 19; the two members of the SIR2 subfamily SbSRT1 and SbSRT2 both contained motif 29. In addition, motif 12 was only present in SbHDA4, SbHDA5, SbHDA8, SbHDA9, and SbHDA12. Thus, it can be inferred that these genes may have some special functions, but the conserved motifs of the entire HDAC family were quite different, which may be due to the different functions of family members.

### Putative Cis-acting regulatory elements in the promoter region of *SbHDACs* genes

**Fig. 3:**
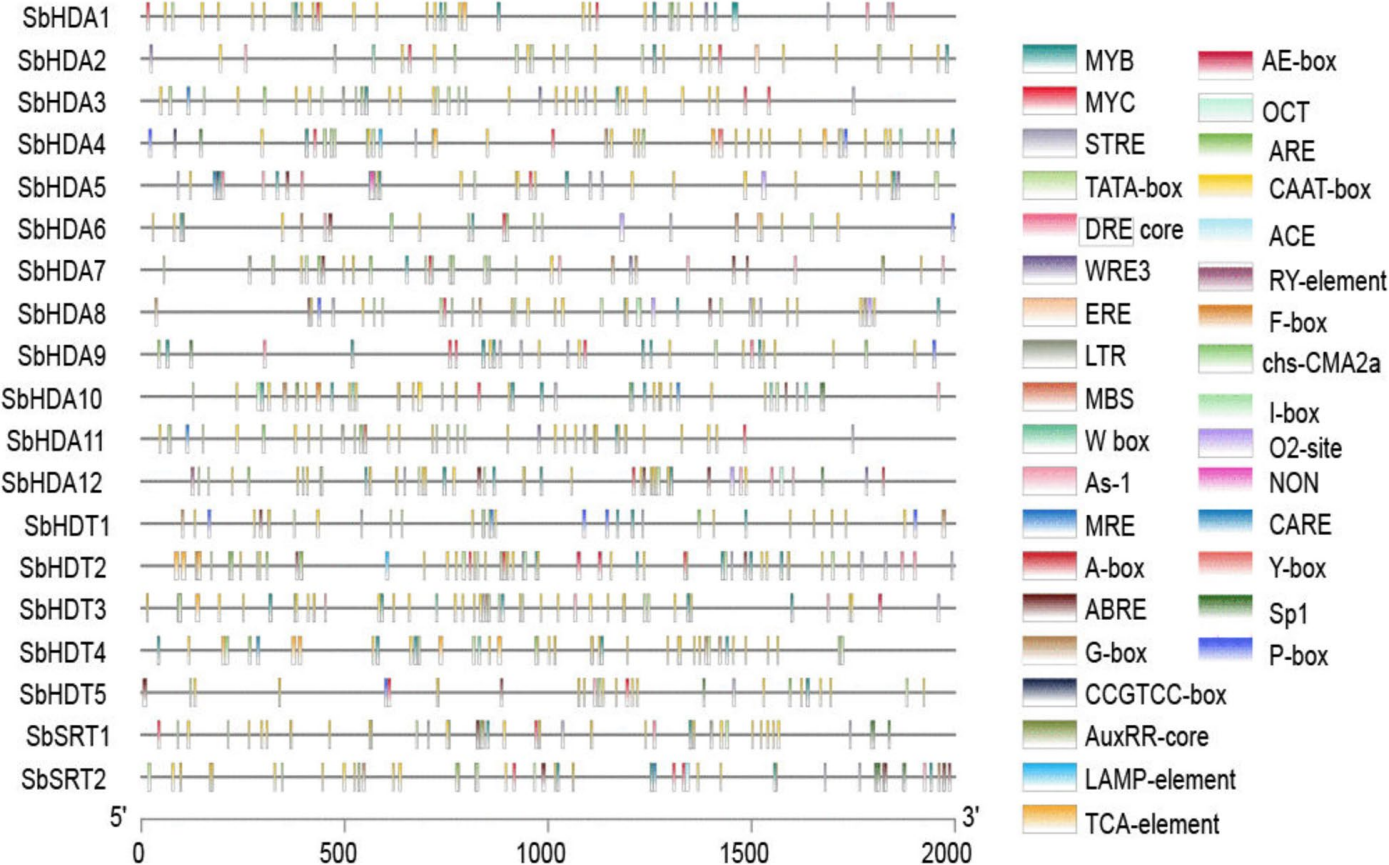
Predicted cis-elements that relate to abiotic stress in the *SbHDACs* promoters. The cis-elements in the 2000 bp upstream promoter regions of *SbHDAC* genes that related to abiotic stress responses are depicted and PlantCARE website is used to predict cis elements. Different cis-elements are represented by different colors

In order to obtain the cis-acting regulatory elements of the *SbHDAC* gene family, we analyzed the sequence of the putative promoter region of each *SbHDAC* gene, and identified 30 putative cis-acting regulatory elements. The results showed that sorghum mainly had the core cis-acting elements TATA and CAAT, as well as some elements related to stress, development, and plant hormone responses such as MYB binding site (MBS), MYC elements (involved in drought tolerance), LTR elements and DRE (involved in low temperature responses), ABRE elements (related to the ABA signaling pathway), P-box elements (related to gibberellin responses), and DRE1 (related to salt stress). The promoter region of each *SbHDAC* gene differed in the type and number of regulatory cis-acting elements (Fig. 3, Table S2). Although RPD3/HDA1, HD2, and SIR2 share most of the cis-regulatory elements in their promoters, some cis-elements are missing in certain subfamilies. For example, LTR cis-acting elements are only present in the promoter regions of *SbHDA2*, *SbHDA3*, *SbHDA7*, and *SbHDA11*. MBS was found in the promoter regions of *SbHDA2*, *SbHDA3*, *SbHDA5*, *SbHDA9*, *SbHDA11*, *SbHDA12*, *SbHDT3*, and *SbSRT2*. The drought response-related element MYB is present in all *HDAC*s. This indicates that different members of the *SbHDAC* gene family may be involved in different abiotic stresses.

### Expression pattern of *SbHDACs* genes in different tissues

**Fig. 4:**
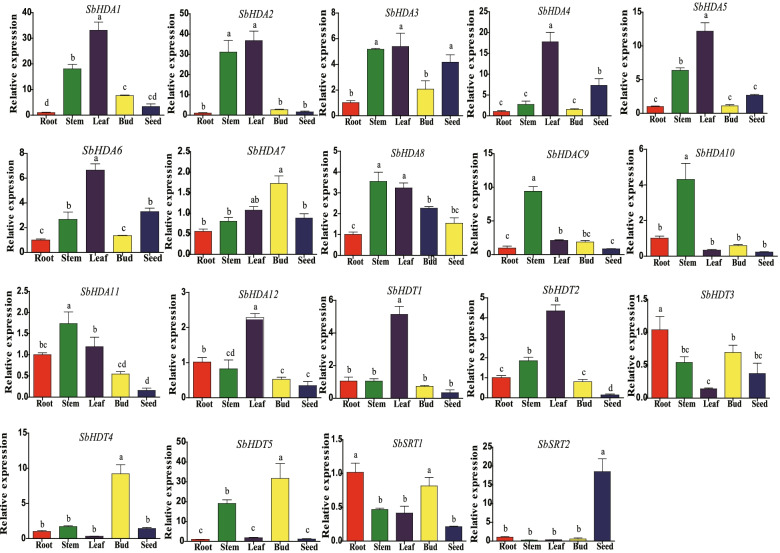
Tissue-specific expression analysis of *SbHDAC* family genes in different sorghum tissues. Different lowercase letters indicate a significant difference determined by the Duncan’s new multiple range test (*P*-value < 0.05); The reference gene is *SbEIF4a*; The y-axis value represents the relative expression

We explored the expression patterns of *SbHDAC* family genes in different tissues including roots, stems, leaves, buds, and seeds. It can be seen from Fig. 4 that these 19 *SbHDAC* genes were successfully detected in five tissues. Nine genes (*SbHDA1*, *SbHDA1*, *SbHDA2*, *SbHDA3*, *SbHDA4*, *SbHDA5*, *SbHDA6*, *SbHDA12*, *SbHDT1,* and *SbHDT2*) were expressed at high levels in leaves, and relatively low levels in roots, stems, leaves, and seeds. *SbHDT3* was expressed in roots, while *SbHDA8*, *SbHDA10*, and *SbHDA11* were highly expressed in stems. *SbHDA7* was most highly expressed in buds. Unlike the other 18 *SbHDAC* genes, *SbSRT2* is expressed in roots and stems, but expression levels in leaves and buds were relatively low and comparable. These results showed that the 19 *SbHDAC* genes were constitutively expressed in five tissues of sorghum, and most were expressed in root, stem and leaf tissues.

### Expression of *SbHDACs* in response to phytohormone

**Fig. 5:**
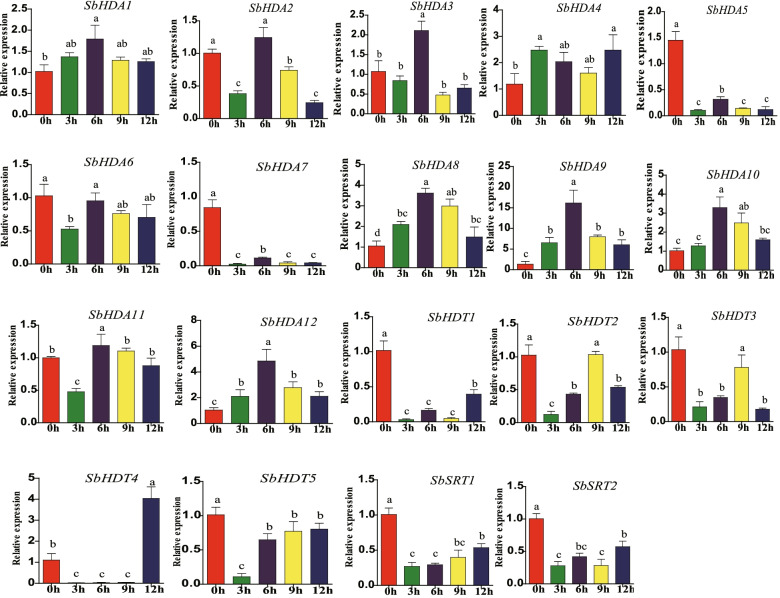
Expression pattern of *SbHDAC* family genes in response to abscisic acid. Different lowercase letters indicate a significant difference determined by the Duncan’s new multiple range test (*P*-value < 0.05); The reference gene is *SbEIF4a*; The y-axis value represents the relative expression and abscisic acid (ABA, 200 μM)

In order to explore the hormone responses of *SbHDAC* gene family members, qRT-PCR was used to analyze the expression patterns of sorghum treated with ABA. The results showed that *SbHDA1*, *SbHDA2*, *SbHDA3*, *SbHDA4*, *SbHDA8*, *SbHDA9*, *SbHDA10*, *SbHDA11*, *SbHDA12*, and *SbHDT4* were significantly induced by ABA, with maximum gene expression levels mainly concentrated at 6 and 12 h for *SbHDA5*, *SbHDA6*, *SbHDA7*, *SbHDT1*, *SbHDT2*, *SbHDT3*, and *SbHDT5* (Fig. 5). Compared with the control group, *SbSRT2* was significantly inhibited under ABA treatment, indicating that ABA could activate and inhibit the expression of different *SbHDACs* genes.

### Expression of *SbHDACs* genes under abiotic stresses

**Fig. 6:**
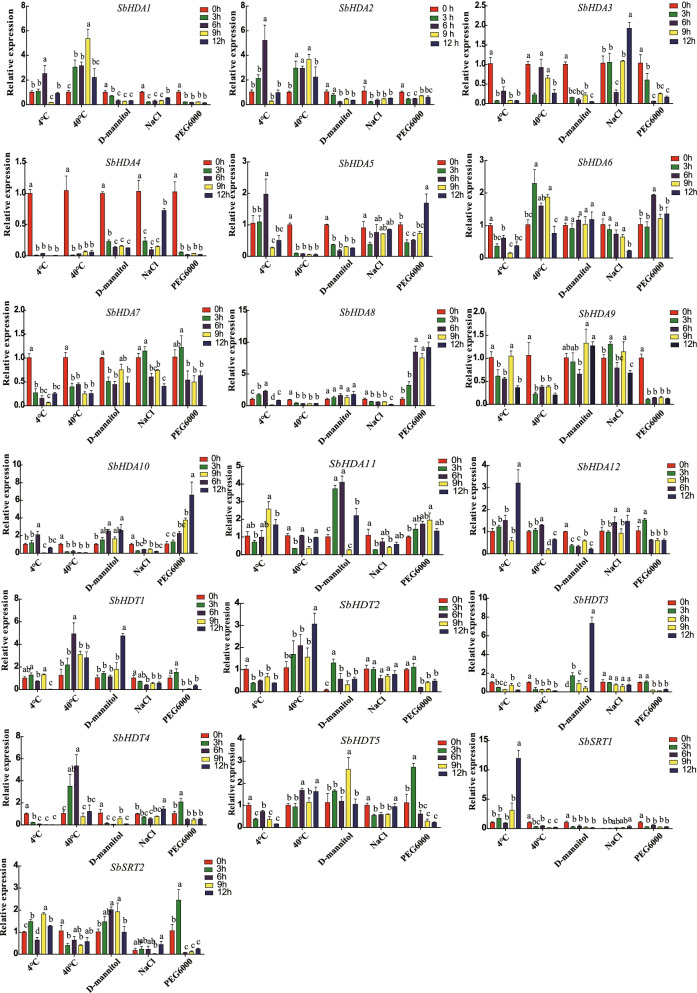
Expression pattern of *SbHDAC* family genes in response to low temperature, high temperature, drought, osmotic and salty stresses. Different lowercase letters indicate a significant difference determined by the Duncan’s new multiple range test (*P*-value < 0.05); The reference gene is *SbEIF4a*; The y-axis value represents the relative expression. Low temperature: 4 °C; High temperature: 40 °C; Drought and osmotic stress: 20% PEG6000 and 300 mM D-mannitol; Salty stress: 250 mM NaCl

Figure 6 shows the expression patterns of *SbHDACs* under low temperature (4 °C), high temperature (40 °C), PEG6000 (drought stress), D-mannitol (osmotic stress), and NaCl stresses (salt stress). In response to low temperature, *SbHDA3*, *SbHDA4*, *SbHDA6*, *SbHDA7*, *SbHDT2*, *SbHDT3*, *SbHDT4*, and *SbHDT5* were significantly inhibited; meanwhile, *SbHDA12* and *SbSRT1* reached maximum expression levels after 12 h of continuous treatment, and the relative expression level of *SbSRT1* was about 12-fold higher than that of the internal control gene, indicating that these two genes respond to low temperature stress. Most of the *SbHDAC* genes in sorghum were significantly inhibited under high temperature, except for *SbHDA1*, *SbHDA2*, *SbHDA6*, *SbHDT1*, *SbHDT2*, and *SbHDT4*. After NaCl treatment, expression of the 19 genes was significantly suppressed, except for *SbHDA3*. Under PEG 6000, D-mannitol drought and osmotic stresses, most genes were suppressed. These results showed that sorghum *HDAC* genes acted differently in response to adversity stresses.

### Expression of *SbHDACs* genes in response to pathogen-associated molecular patterns (PAMPs)

**Fig. 7:**
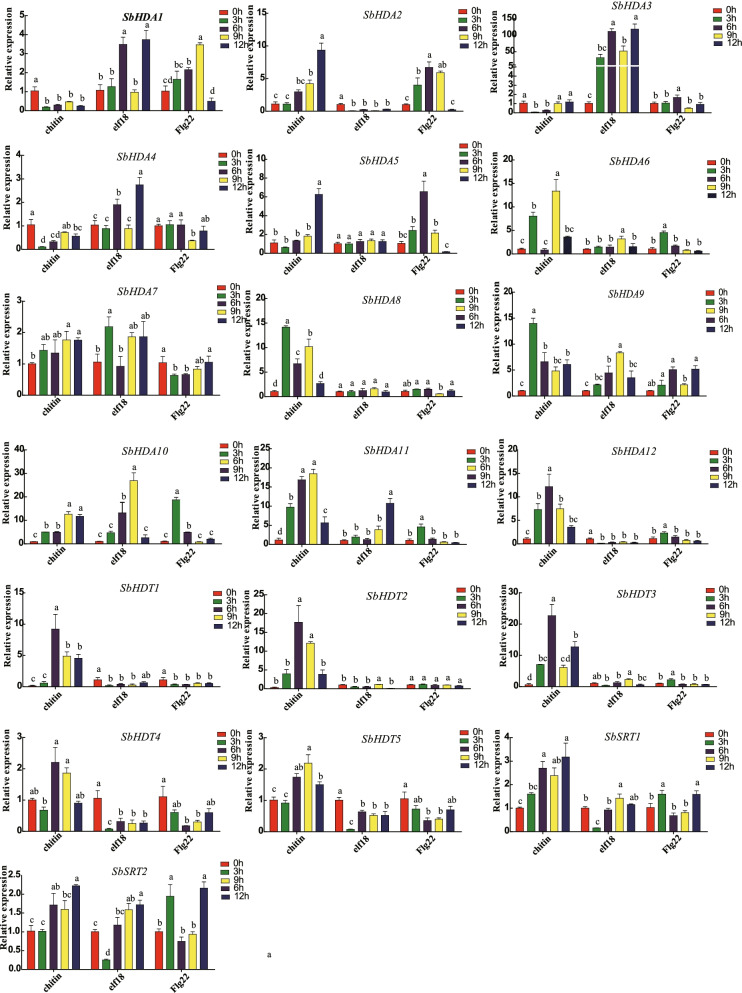
Expression pattern of *SbHDAC* family genes in response to PAMPs. Different lowercase letters indicate a significant difference determined by the Duncan’s new multiple range test (*P*-value < 0.05); The reference gene is *SbEIF4a*; The y-axis value represents the relative expression. Flagellin (flg22, 100 nM), translation elongation factor (elf18, 100 nM) and chitin (chitin, 8 nM)

PAMP elicitors chitin, flg22, and elf18 were used to simulate biological stress, and the expression of *SbHDACs* was measured to evaluate the effects under PAMPs stress (Fig. 7). It was found that after chitin treatment, expression of *SbHDA8* and *SbHDA9* genes was increased significantly, and reached a maximum at 3 h. Expression of *SbHDA12*, *SbHDT1*, *SbHDT2*, *SbHDT3*, and *SbHDT4* initially increases, then decreased, and reached to the peak at 6 h. *SbHDA2*, *SbHDA5*, *SbSRT1*, and *SbSRT2* reached maximum expression levels after 12 h of continuous treatment, indicating that they were relatively slow in responding to chitin treatment. By contrast, after chitin treatment, expression of *SbHDA1*, *SbHDA3*, and *SbHDA4* was suppressed. Meanwhile, *SbHDA3*, *SbHDA4*, *SbHDA7*, *SbHDA9*, *SbHDA10*, *SbHDA11*, *SbSRT1*, and *SbSRT2* were significantly induced by elf18. Under flg22 treatment, the expression patterns of most *HDACs* revealed an initial increase followed by a decrease in expression level. The above results indicated that chitin, flg22, and elf18 could activate and inhibit the expression of *SbHDACs*, which implied that *SbHDACs* were involved in different plant innate immune response processes.

### Functional verification of SbHDACs: prokaryotic expression and spot assays of *E. coli* expressing *SbHDA1*, *SbHDA3*, *SbHDT3*, and *SbSRT2* under abiotic stresses

**Fig. 8:**
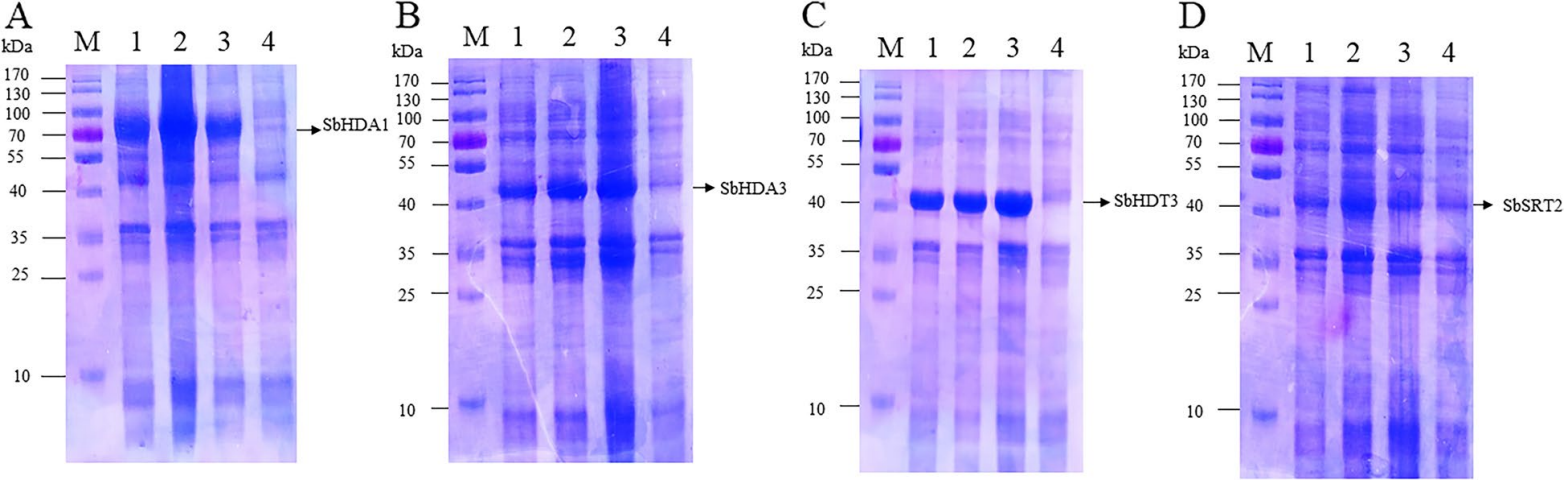
Prokaryotic expression analysis of SbHDA1, SbHDA3, SbHDT3 and SbSRT2 protein at different temperatures. M: Protein marker; Lane 1-3: Supernatant from SbHDA1, SbHDA3, SbHDT3 and SbSRT2 culture after IPTG (1.0 mM) induction at 16 °C, 25 °C and 30 °C for 24 h, 16 h and 12 h, respectively; Lane 4: Supernatant of empty vector

**Fig. 9:**
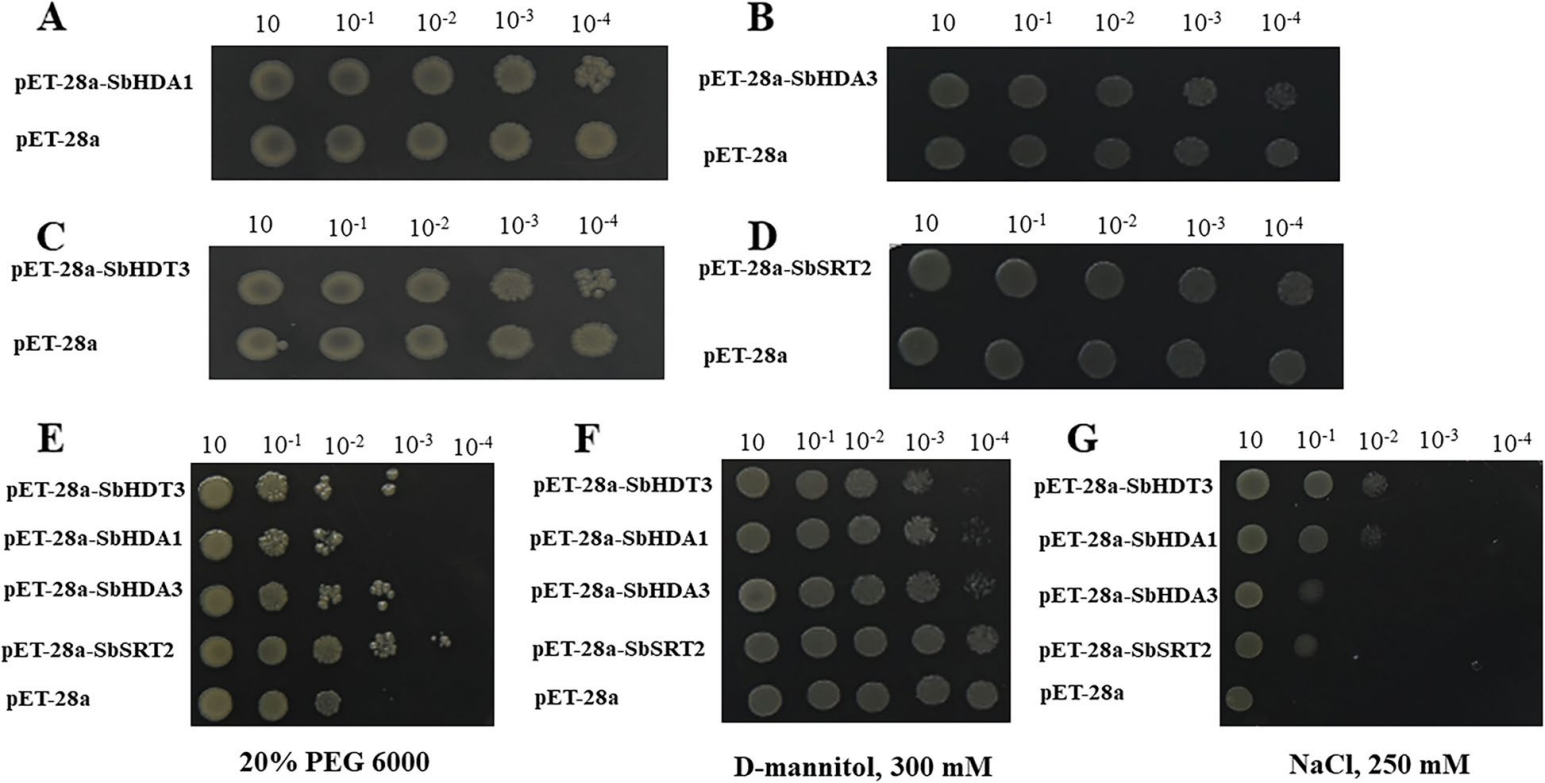
Spot assay of Rosetta (DE3) is transformed with plasmids. **A**-**D** represent pET28a-*SbHDA1*, pET28a-*SbHDA3*, pET28a-*SbHDT3* and pET28a-*SbSRT2* and pET-28a plasmid under normal condition (37 °C), respectively. **E**-**G** represent the growth of recombinant strains under PEG 6000 (20%), NaCl (250 mM) and D-mannitol (300 mM) treatments at 37 °C

To confirm the function of SbHDACs, *SbHDA1*, *SbHDA3*, *SbHDT3* and *SbSRT2* were amplified and inserted into pET28a for prokaryotic expression (Fig. S5). The coomassie brilliant blue staining results showed that SbHDA1, SbHDA3, SbHDT3, and SbSRT2 proteins were solubly expressed in *E. coli* when induced at 16 °C, 25 °C or 30 °C (Fig. 8), and western blot results showed that the expressed protein is HDAC proteins (Fig. S6). Subsequentially, the growth of recombinant bacteria (with pET-28a-SbHDA1, SbHDA3, SbHDT3 or SbSRT2) and control (pET-28a) were further examined under different stresses (PEG 6000, NaCl and D-mannitol) on the LB medium (Fig. 9). The spot growth results showed that after 12 h of culture, bacteria harboring *SbHDA1*, *SbHDA3*, *SbHDT3* or *SbSRT2* gene showed higher growth rate than with empty vector under drought (20% PEG 6000) and salt stress (NaCl) (Fig. 9E-G). Meanwhile, all the recombinant bacteria showed the same growth rate under normal condition (Fig. 9A-D). However, under D-mannitol stress, the growth rate of recombinant bacteria was almost insignificant compared with control (Fig. 9F). All the results demonstrated that *SbHDA1*, *SbHDA3*, *SbHDT3* and *SbSRT2* may contribute the tolerance of *E. coli* under PEG 6000 and NaCl stress.

### Sorghum histone acetylation level are upregulated under stresses treatment

**Fig. 10:**
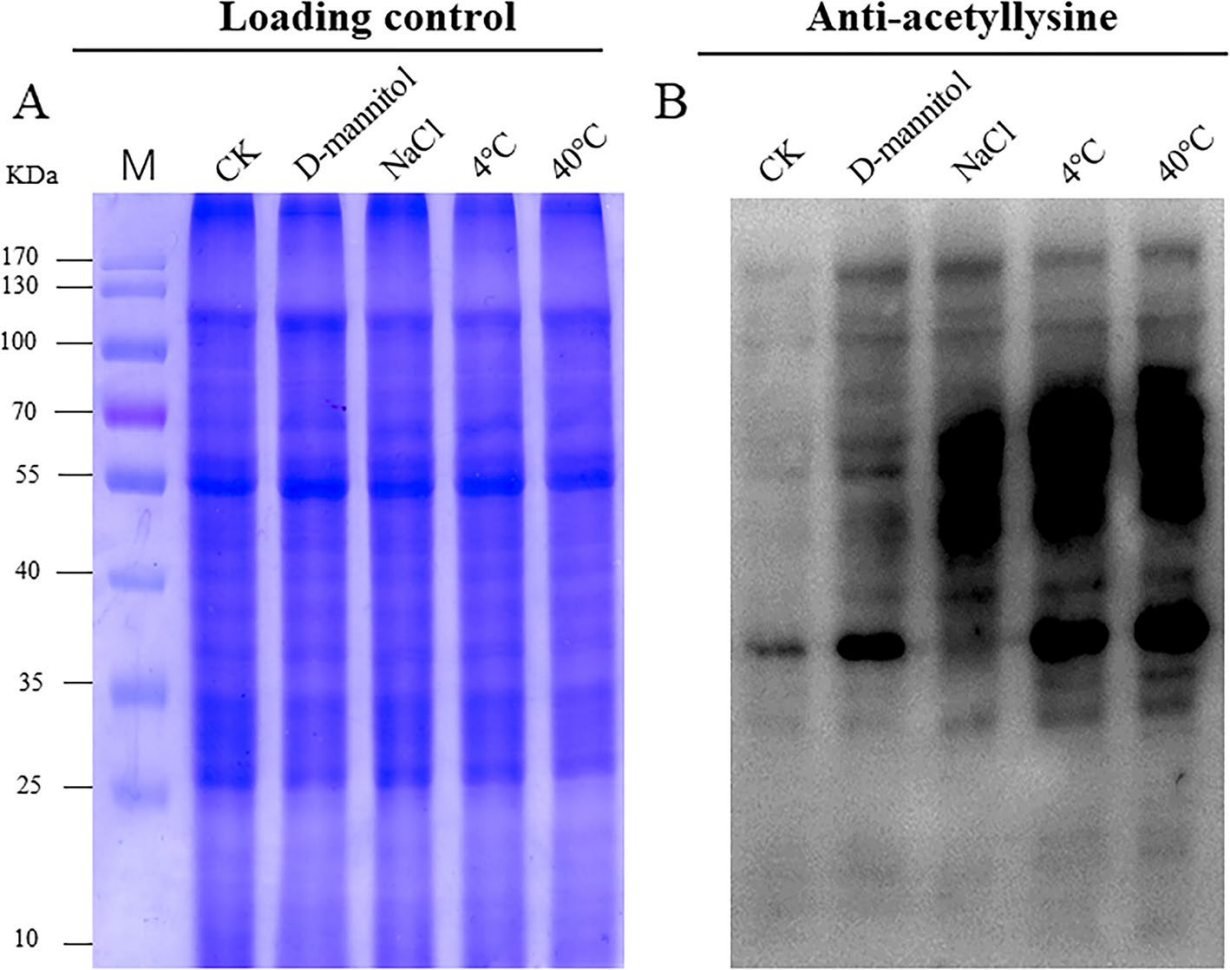
Acetylation level of sorghum under stress treatments. **A** The SDS-PAGE gel was stained with Coomassie Brilliant Blue (CBB) as the loading control. Three-leaves stage seedlings were treated under cold (4 °C), heat (40 °C), osmotic stress (300 mM D-mannitol) and salt (250 mM NaCl) stresses for 12 h. **B** Western blot showing the acetylation level in sorghum treated under cold, heat, osmotic stress and salt stresses, CK: Water-treated sorghum seedlings

Additionally, Pan-acetyl lysine antibody was used to examine the acetylation level of sorghum in response to different stresses (cold, heat, osmotic and salt stresses). As shown in Fig. 10, multiple lysine acetylated protein bands were detected in all samples. However, compared with the control group, stronger acetylation level was observed following the stress treatments (Fig. 10B), while all the proteins were loaded in the same amount (Fig. 10A).

## Discussion

Epigenetics mainly refers to the study of heritable gene expression changes that do not involve changes in DNA sequence. It plays a very important role in the growth and development of plants, and histone modification is closely related to gene expression regulation. HDACs, also called lysine deacetylases, are enzymes that regulate gene expression by removing acetyl groups from core histones (H2A, H2B, H3, and H4) [36]. Studies have shown that HDACs play a key role in plant growth and development [7], including genome stability [3], and responses to various environmental stresses [5]. Members of the HDAC family have been widely reported in a variety of plants. However, research on *HDAC* genes in sorghum is scarce. Therefore, in this study we conducted a comprehensive genomic analysis of the sorghum *SbHDAC* gene family, and the results provided a strong theoretical basis for future functional studies.

The involvement of *HDACs* in the responses to environmental cues has not been documented in sorghum. Herein, 19 *HDACs* were identified in the sorghum genome, and characterized in terms of tissue-specific expression profiles, biotic and abiotic stress response expression patterns, prokaryotic expression, and acetylation levels. The 19 SbHDACs belong to three subfamilies: RPD3/HDA1, SIR2, and HD2. The number of *SIR2* subfamilies in sorghum is similar to that in *Arabidopsis*, rice, and tomato, all of which have two *SIR2* genes, while soybean contains four *SIR2* genes [37]. All members of the RPD3/HDA1 subfamily have a conserved HDAC domain, while members of the SIR2 and HD2 subfamilies have SIR2 and C2H2 Zinc finger domains, similar to those reported in *Arabidopsis* [23], *Oryza sativa* [24], and *Zea mays* [38]. Genes belonging to the same subfamily may share similar structures. All *SbHDACs* contain a variety of conserved motifs that are highly similar to those in other plants, which strongly indicates that *SbHDACs* may have similar functions to homologous genes.

Previous reports showed that RPD3 type Class I *HDACs* are localized exclusively in the nucleus in humans, whereas class II *HDACs* are shuttled between the cytoplasm and the nucleus [39]. In *Arabidopsis*, previous studies demonstrated that RPD3 type Class II *HDACs HDA5*, *HDA8*, and *HDA14* are localized in the cytoplasm, whereas *HDA15* is localized exclusively in the nucleus. In addition, *AtHDA15* was shown to shuttle from the cytoplasm to the nucleus in response to light [18]. In soybean [37], *GmHDA6*, *GmHDA13*, *GmHDA14*, and *GmHDA16* are located in the nucleus and cytoplasm, while in sorghum, *SbHDA8*, *SbHDA9*, and *SbHDA12* are also located in the nucleus and cytoplasm, and *SbHDA2*, *SbHDA6*, and *SbHDA11* are also located in the chloroplast and nucleus, suggesting a possible shuttling process between these compartments. The two members of the soybean HD2 subfamily are localized in the nucleus, while in the present study, five members of the sorghum HD2 subfamily were found to be localized in the nucleus. SIR2 proteins are reported to occupy discrete subcellular compartments in plants. For example, in rice, OsSRT1 is found in the nucleus [40], and *OsSRT2* and *AtSRT2* are found in mitochondria [41], but *SlSRT2* is localized in both the nucleus and cytoplasm [42]. In the present study, we predicted that *SbSRT1* and *SbSRT2* are localized in the chloroplast. Overall, the subcellular localization patterns of different genes in sorghum indicates that they might be differentially regulated and may have distinct roles.

Increasing evidence indicates that responses of *HDACs* to the environment stress play a key role in plant growth and development [5]. For example, in *Arabidopsis*, the AtHDAC protein reportedly participates in responses to environmental stresses such as salt, drought, and temperature. It also participates in seed development, senescence, embryonic development, photomorphogenesis, senescence, and flowering processes [43–45]. In rice, *HDACs* are involved in flowering [46], root development [47], seed germination and responses to environmental stress [48]. In tomato, *SlHDA1*, *SlHDA3*, *SlHDA4*, and *SlHDA5* are involved in the response to different abiotic stresses [49]. The *PtHDT902* gene in poplar has a strong influence on the formation of the root system, and salt tolerance of poplar has a negative regulatory role [50]; in barley, the *HDA1* gene plays a vital role in development and epigenetic regulation [51]. In sorghum, *SbHDAC* genes belonging to RPD3/HDA1, HD2, and SIR2 groups displayed higher transcription levels in five different tissues. However, in tea plants, only RPD3/HDA1 and HD2 subfamily genes were expressed at high levels in different tissues, while SIR2 subfamily transcription levels were very low, indicating that *HDAC* genes are expressed differently in species. Under salt, simulated drought, and temperature stress, the expression of most of genes in sorghum was suppressed, consistent with the results reported in *Arabidopsis* [30], barley [52], and rice. In sorghum, *SbHDA3* was up-regulated, and research on *Arabidopsis* indicates that overexpression of *CsHD2C* can enhance the sensitivity to ABA and NaCl stress, but whether *HDA3* has the same function in sorghum requires further verification. In addition, under treatment with exogenous ABA, the expression of most *SbHDAC* genes was up-regulated, similar previous results in tea plants [27]. In our current study, *SbHDA1*, *SbHDA3*, and *SbHDA8* were down-regulated following NaCl treatment and induced by application of ABA, consistent with previous findings. In addition, the NtHD2s helps tobacco to improve the adaptability against salt stress [53]; In rice [32], through yeast two-hybrid screening analysis, it was found that HDA705 can interact with Hsf B1 family protein (RHSF10), and salt responsive WD40 protein (SRWD) to regulate stress response. The above results indicate that sorghum *SbHDACs* could respond to different abiotic stresses.

## Conclusions

In present study, we identified 19 *HDACs* genes from *Sorghum bicolor*, which were divided into three subfamilies: RPD3/HDA1, SIR2 and HD2. The cis-acting elements and real-time PCR results indicate that *HDACs* genes play important roles in participating in stress resistance. Furthermore, sorghum has undergone significant changes in its acetylation level under adversity treatments. Therefore, our research provides help to understand the *HDACs* gene of sorghum and lay a solid foundation for the improvement of other crops.

## Methods

### Plant materials, growth conditions, and stress treatments

After surface disinfection, the germinated sorghum BTx623 seeds were planted in sterilized soil (Pindstrup, Denmark) and cultivated in a greenhouse at 25/20 °C under a 14 h light/10 h dark cycle. When the seedlings grow to the three-leaf stage, abscisic acid (ABA, 200 μM), 20% PEG 6000, mannitol (D-mannitol, 300 mM) and sodium chloride (NaCl, 250 mM) were sprayed to the seedlings separately. For temperature treatments, sorghum seedlings were kept in a constant temperature incubator at 4 °C or 40 °C, and 25 °C was as the control. In response to biological stresses, the seedlings were sprayed with PAMPs such as flg22 (100 nM), elf18 (100 nM) or chitin (8 nM); samples were collected at 0, 3, 6, 9 and 12 h post treatments. Sorghum tissues (roots, stems, leaves, buds and seeds) were sampled under normal condition. All the samples, three biological replicates were set up, and three seedlings were processed in each replicate. The samples were quickly frozen with liquid nitrogen and stored in a refrigerator at − 80 °C for further use in RNA extraction. All methods were performed in accordance with the protocols set up based on the relevant guidelines and regulations.

### Identification of *HDAC* genes in *Sorghum bicolor*

The whole genome sequence of sorghum was downloaded from NCBI (https://www.ncbi.nlm.nih.gov/) as the local database. The hidden Markov model (HMM) configuration files PF00850 (Hist_deacetyl domain), PF02146 (SIR2 domain) and PF17800 (NPL domain) of the HDAC family were extracted from the Pfam database (http://pfam.sanger.ac.uk). Then, the HMM configuration files were used to search for target sequences with conserved domains in the local sorghum protein database through HMMER 3.0 (http://hmmer.janelia.org/) with the E-value≤1 × 10^− 5^. The basic physical and chemical properties of sorghum SbHDAC family proteins are predicted with ExPASy-ProtParam tool (https://web.expasy.org/protscale/). Protein subcellular localization was predicted through Cell-PLoc 2.0 (http://www.csbio.sjtu.edu.cn/cgi-bin/PlantmPLoc.cgi). Chromosome location and gene structure were separately performed by MG2C (http://mg2c.iask.in/mg2c_v2.0/) and GSDS (http://gsds.cbi. pku.edu.cn/). MEME online software (http://meme-suite.org/tools/meme) was used to analyze conservative motif with the motif parameters at 30, and the rest are default.

### Phylogenetic tree construction and sequence alignment

The HDAC protein sequences of *Arabidopsis*, rice and tomato were downloaded from TAIR (https://www.arabidopsis.org/) and NCBI databases, and MEGA 7.0 software was used to construct a phylogenetic tree of *HDAC* gene family by the neighbor-joining method (Neighbor-Joining, NJ; bootstrap = 1000), and DNAMAN 7.0 software was used to align the amino acid sequences.

### Collinearity analysis of sorghum *HDAC* gene family

Sorghum HDAC collinearity was identified by MCScanX verson 1.0, and TBtools (v1.05) [54] was used to drawn the Fig. 2.

### Characterization of *SbHDAC* genes and proteins

Based on CDS sequence, sorghum interspecific phylogenetic tree and genome sequence, TBtools v1.05 was used to predict the number of introns/exons of *SbHDACs* [54]. MEME online program was used to analyze the conserved motif structure of the SbHDAC protein. To predict cis-acting elements of sorghum *SbHDAC* genes, the 2000 bp upstream sequences of the sorghum transcription start site from the NCBI database were extracted for analyzing through PlantCARE (http://bioinformatics.psb.ugent.be/webtools/plantcare/html/).

### Total RNA extraction and qRT-PCR analysis

The total RNA was extracted by TRIzol, and the integrity of the extracted total RNA was examined by 1.0% agarose gel electrophoresis. cDNA was synthesized using HiScript® III RT SuperMix (Vazyme Biotech Co. Ltd.). Bio-Rad real-time PCR system (Bio-Rad, Hercules, CA, USA) was used to perform qRT-PCR expression analysis, and sorghum *SbEIF4a* was used as an internal reference gene. The primer pairs used for qRT-PCR were listed in Table S1. After the amplification is completed, the melting curve and amplification curve were checked to evaluate the amplification specificity. All experiments were repeated three times for each sample. Fluorescence quantitative data was analyzed by the 2^-ΔΔCt^ [55], and the Duncan’s new multiple range test (based on SPSS software) was employed for significance analysis.

### Protein prokaryotic expression in *E. coli*

The ORF of *SbHDA1*, *SbHDA3*, *SbHDT3* and *SbSRT2* was amplified and inserted into pET28a (+) vector respectively. The obtained recombinant plasmid was transformed into *E. coli* Rosetta (DE3) competent cells. 1.0 mM isopropyl *β*-D-thiogalactoside (IPTG) was used to induce protein expression at 16 °C, 25 °C and 30 °C for 24 h, 16 h and 12 h, respectively [56]. The prokaryotic protein was evaluated through 12% SDS-PAGE electrophoresis by coomassie brilliant blue staining. Western blot was used to confirm the size of expressed protein to be HDAC. In order to study the expression of SbHDA1, SbHDA3, SbHDT3 and SbSRT2 in *E. coli* under different abiotic conditions, a spot assay was conducted in combination with treatment using PEG6000, D-mannitol and sodium chloride. The *E. coli* cells containing pET28a-*SbHDA1*, *SbHDA3*, *SbHDT3* and *SbSRT2* or pET28a (control) were cultured in LB medium at 37 °C until the OD_600_ reaches to 0.6. Then, 1.0 mM IPTG was added and further cultured at 37 °C for 12 h. Then LB medium (Composition of the medium are tryptone, yeast extract, NaCl and agar) was used to dilute the cultures to 10 ~ 10^− 4^ times. All treatments were cultured at 37 °C overnight for photographing.

### Protein extraction and immunoblot analysis

The sorghum seedlings were treated by 4 °C, 40 °C, 300 mM D-mannitol and 250 mM NaCl for 12 h, and then the samples including the control were quickly ground in liquid nitrogen. Total protein was extracted by lysis buffer (50 mM Tris-HCl pH 7.5, 150 mM NaCl, 0.1% NP-40, 8 M urea, 1 mM PMSF, 1 × cocktail, 50 mM nicotinamide, 3 μM Trichostatin A). After adding loading buffer and boiling for 5 min (100 °C), the protein was separated by 12% sodium dodecyl sulfate polyacrylamide gel electrophoresis (SDS-PAGE), then transferred to polyvinylidene fluoride membrane (PVDF) membrane. Finally, the acetylated protein was examined with anti-acetyl lysine antibody (1:1000 dilution, PTM Biolabs, Hangzhou, China), The secondary antibody was goat anti-mouse antibody (1:10000 dilution).

## Supplementary Information

**Additional file 1: Figure S1.** Chromosome distribution of *SbHDAC* genes in sorghum.

**Additional file 2: Figure S2.** Multiple sequence alignment of the SbHDAC proteins.

**Additional file 3: Figure S3.** Phylogenetic relationships and gene structures of the *SbHDAC* family. Exons and introns were shown by filled boxes and single lines, respectively.

**Additional file 4: Figure S4.** Motif analysis of SbHDAC proteins.

**Additional file 5: Figure S5.** Amplification and plasmids construction of *SbHDAC* genes into pET28a. A-D: lane 1, recombinant plasmids. lane 2, double digestion of *SbHDA1-*pET28a, *SbHDA3-*pET28a, *SbHDT3-*pET28a, and *SbSRT2-*pET28a respectively.

**Additional file 6: Figure S6.** Detection of SbHDA1, SbHDA3, SbHDT3 and SbSRT2 protein by western blot. M: Protein marker; Lane1: Supernatant of empty vector; Lane 2: Supernatant of SbHDA1, SbHDA3, SbHDT3 and SbSRT2 recombinant cells.

**Additional file 7: Table S1.** Primer sequences.

**Additional file 8: Table S2.** Cis-acting element analysis of the *SbHDAC* gene family.

## Data Availability

All data generated or analyzed during this study are included in this published article and its supplementary information files.

## Abbreviations

HDACs: Histone deacetylases
PAMPs: Pathogen-associated molecular model
HATs: Histone acetyltransferases
CBP: CREB binding protein
NAD^+^: Nicotinamide adenine dinucleotide
ABA: Abscisic acid
JA: Jasmonic acid
PR1: Pathogenesis Related 1
SA: Salicylic acid
Xoo: *Xanthomonas oryzae* pv *oryzae*
ROS: Reactive oxygen species
*E.coil*: *Escherichia coli*
IPTG: Isopropyl β-D-thiogalactoside
SDS-PAGE: Sodium dodecyl sulfate polyacrylamide gel electrophoresis
PVDF: Polyvinylidene fluoride
qRT-PCR: Real-time fluorescence quantitative PCR
ABRE: Abscisic acid responsiveness elements
MYB: v-myb avian myeloblastosis viral oncogene homolog
MYC: Myelocytomatosis protein
DRE: Dehydration-responsive element
ABRE: ABA responsive element
