# Supplementary material for: Characterization of histone deacetylases and their roles in response to abiotic and PAMPs stresses in Sorghum bicolor
Source: BMC Genomics. 2022 Jan 6;23:28. doi: 10.1186/s12864-021-08229-2 (PMC8739980; doi:10.1186/s12864-021-08229-2)
Supplement: Supplementary file 1 — Additional file 1: Figure S1. Chromosome distribution of SbHDAC genes in sorghum. [file 12864_2021_8229_MOESM1_ESM.pdf]

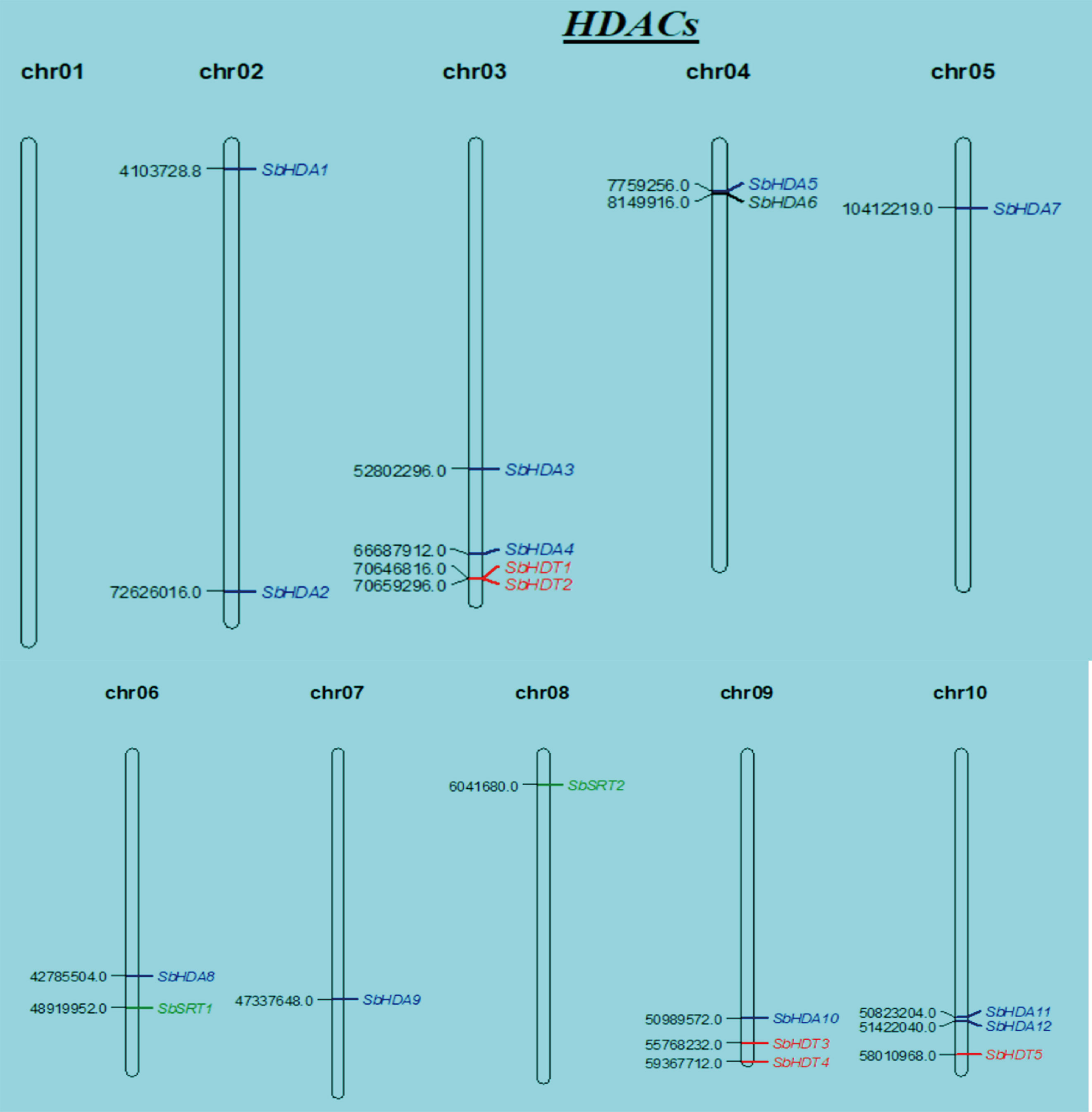
HDACs
chr01
chr02
chr03
chr04
chr05
4103728.8
SbHDA1
72626016.0
SbHDA2
52802296.0
SbHDA3
66687912.0
SbHDA4
70646816.0
SbHDT1
70659296.0
SbHDT2
7759256.0
SbHDA5
8149916.0
SbHDA6
10412219.0
SbHDA7
chr06
chr07
chr08
chr09
chr10
42785504.0
SbHDA8
48919952.0
SbSRT1
47337648.0
SbHDA9
6041680.0
SbSRT2
50989572.0
SbHDA10
55768232.0
SbHDT3
59367712.0
SbHDT4
50823204.0
SbHDA11
51422040.0
SbHDA12
58010968.0
SbHDT5
