# Supplementary material for: Characterization of histone deacetylases and their roles in response to abiotic and PAMPs stresses in Sorghum bicolor
Source: BMC Genomics. 2022 Jan 6;23:28. doi: 10.1186/s12864-021-08229-2 (PMC8739980; doi:10.1186/s12864-021-08229-2)
Supplement: Supplementary file 2 — Additional file 2: Figure S2. Multiple sequence alignment of the SbHDAC proteins. [file 12864_2021_8229_MOESM2_ESM.pdf]

```
SbHDA1     MASDMRAPSSGKKSETSHGMKCGVSDQACHGKCQSCDIAA   40
SbHDA2     ........................................    0
SbHDA3     ........................................    0
SbHDA4     ........................................    0
SbHDA5     ........................................    0
SbHDA6     ........................................    0
SbHDA7     ........................................    0
SbHDA8     ........................................    0
SbHDA9     ........................................    0
SbHDA10    ........................................    0
SbHDA11    ........................................    0
SbHDA12    ........................................    0
SbHDT1     ........................................    0
SbHDT2     ........................................    0
SbHDT3     ........................................    0
SbHDT4     ........................................    0
SbHDT5     ..MGGSGGGAGQRSGAHLTHAGAEGENAYWRRRDQYREAN   38
SbSRT1     ........................................    0
SbSRT2     ........................................    0
Consensus

SbHDA1     KPICSGVDGVSSLTGSHTDVKASKENCGACSLNNDRADSL   80
SbHDA2     ........................................    0
SbHDA3     ........................................    0
SbHDA4     ........................................    0
SbHDA5     ........................................    0
SbHDA6     ........................................    0
SbHDA7     ........................................    0
SbHDA8     ........................................    0
SbHDA9     ........................................    0
SbHDA10    ........................................    0
SbHDA11    ........................................    0
SbHDA12    ........................................    0
SbHDT1     ........................................    0
SbHDT2     ........................................    0
SbHDT3     ........................................    0
SbHDT4     ........................................    0
SbHDT5     ESGGLVGAGWQEARQPAGKPEAGGARAAVPSSPVCECVAV   78
SbSRT1     ........................................    0
SbSRT2     ........................................    0
Consensus

SbHDA1     EEEVKGSTARIGHVESADPDGCVDVKKESFMAVDDLPQEF  120
SbHDA2     ........................................    0
SbHDA3     ........................................    0
SbHDA4     ..............................MSPLFRHCCA   10
SbHDA5     ........................................    0
SbHDA6     ........................................    0
SbHDA7     ........................................    0
SbHDA8     ........................................    0
SbHDA9     ........................................    0
SbHDA10    ........................................    0
SbHDA11    ........................................    0
SbHDA12    ........................................    0
SbHDT1     ........................................    0
SbHDT2     ........................................    0
SbHDT3     ........................................    0
SbHDT4     ........................................    0
SbHDT5     SSSAPREAGRSTRFGCSHQPRNAGSPLGQSRIRIPKPTLG  118
SbSRT1     ........................................    0
SbSRT2     ........................................    0
Consensus

SbHDA1     EGEQVGATLEDLFFFNGEEEDDSDWEPASRLVEDRWFCFN  160
SbHDA2     .................................MSAPAPA    7
SbHDA3     ................................MDTSGNSL    8
SbHDA4     FGSHHPHPLIWRSIFQTSGREQKQIRSWRAQCSANQDQAS   50
SbHDA5     ..............................MDPSSAGSGG   10
SbHDA6     ........................MASTSG..DTSAAEAL   14
SbHDA7     ..........................MGKRRSEHPQAPPL   14
SbHDA8     ........................................    0
SbHDA9     ..............................MAAS.GEGAS    9
SbHDA10    ..........................MASESSPPPAEAPP   14
SbHDA11    ........................MASSSSPAPTLAGEAL   16
SbHDA12    ..............................MDAS.AGGGG    9
SbHDT1     ........................................    0
SbHDT2     ........................................    0
SbHDT3     ........................................    0
SbHDT4     ........................................    0
SbHDT5     ITLLLVSGTGTAPSASLTSRSQKPRTLSSREAPRRREAAG  158
SbSRT1     .....................MSLGYAEKLSYREDVGTVG   19
SbSRT2     ...............................MLADTSAAH    9
Consensus

SbHDA1     CTMPIVDQITHCMNCRELKESAVDGYDVFKK.QIAQTALL  199
SbHDA2     PAPPMAAPRVG...........LLYDDRMRAHATPDGEEH   36
SbHDA3     PSPSCP..DG.......KKREVCYYYDAGIA.SVDYGEDH   38
SbHDA4     IEKSNAPKNLAIPDDSLLDARILYCTSPALG...HNKEAH   87
SbHDA5     NSLPSVGADG.......QKRRVCYFYDSDVG.NYYYGQGH   42
SbHDA6     RRGRILSSRLYLDGVPSSKAPVVYSPAYDIE.FNGIEKQH   53
SbHDA7     PPEPCAGDDGGGG..VTKQKSVCYYTTTHASPTLTTARQH   52
SbHDA8     ........ML.......EKDRISYFYDGDVG.NVYFGPNH   24
SbHDA9     LPSPAGGEDA.......HRRRVSYFYEPSIG.DYYYGQGH   41
SbHDA10    PVPGNKLAVFWHEGMLAHDAGRGVFDSGRDPGFLDVLDQH   54
SbHDA11    RQKRILSSKLYLE.VPSSKAPVVYSPAYDIS.FLGLEKLH   54
SbHDA12    NSLPTTGADG.......SKRRVCYFYDAEVG.NYYYGQGH   41
SbHDT1     ...........MGIEMPQCG....STGVVVR.PGETVKCD   24
SbHDT2     ......................MEFWGKEVK.PGATVSCE   17
SbHDT3     ......................MEFWGLEVK.PGSTVKCE   17
SbHDT4     ......................MEFWGLEVK.PGSTVKCE   17
SbHDT5     RRSRTPSRALLPRRAMPMVNGIFEYWGVVVR.PGATVKCD  197
SbSRT1     MPEIFEPPELVQNKIEELAAMVQKSKHLVVFTGAGISTSS   59
SbSRT2     VTADVARHGSCWCWSRHFPPFVYTSHQTKEEPAPAACLPR   49
Consensus

SbHDA1     SADTELLPVSTAIGFDERMLLHSEQLEVKPNPHPE.RPDR  238
SbHDA2     PENPERLRAIWRKLNAEGVAPRCVALKAK.........EA   67
SbHDA3     SMVPRRVDMAHALVRSYGLLHDMRRLRTR........PAT   70
SbHDA4     PESNKRVPAIVDALEKLELSPKHRGSQVLEIQN...FNPA  124
SbHDA5     PMKPHRIRMTHSLLARYGLLNQMQVYRPN.........PA   73
SbHDA6     PFDSAKWGRVRNFLVDAGLLQNDRIIEPL.........EA   84
SbHDA7     EMVPHRVSMTHALINAYGRLARRHGPSPRRAGHRARPPAS   92
SbHDA8     PMKPHRLCMTHHLVLSYGLHKKMEIYRPH.........KA   55
SbHDA9     PMKPHRIRMAHSLVVHYGLHRLLELSRPY.........PA   72
SbHDA10    PENADRVRNMVSILRRGPIAHFLSWHSGR.........PA   85
SbHDA11    PFDSAKWGRICRYLTREGYLDKKRMVDPL.........EA   85
SbHDA12    PMKPHRIRMTHALLGRYGLLDQMQVLRPH.........PA   72
SbHDT1     PGD.SFCHISQIALQAGKG.NEDVRIFMK........VDG   54
SbHDT2     VGNDLVIHLSQAALGEPKKASENVIVSVK........IDD   49
SbHDT3     PGHGFILHLSQAALGESKK.SDNALMYVK........VDD   48
SbHDT4     PGHGFILHLSQAALGESKK.SDSVLMYVK........VDD   48
SbHDT5     PGE.FYCHVSQIALQDSKG.NEDVRVFVK........ADG  227
SbSRT1     GIPDFRGPKGVWTLQRAGKGVPNASLPFHR.....AVPSL   94
SbSRT2     VLRTTRLHVTFAAARPPAGARRRRPMAAAP....AAHAYR   85
Consensus

SbHDA1     LRAIAASLAAAGIFPSKCALVPPREITKEELLMVHSPDHI  278
SbHDA2     EDKYIASVHSKSHIKLMKEIS.SKKYDATRNKIARKFNSI  106
SbHDA3     VTEITGDFHEPDYVRLLQNLTPESFYAGGATMSAAKAHNI  110
SbHDA4     SLDDVARVHSRSYITGLEKAMRRASDEGLIFIEGTGPTYA  164
SbHDA5     RDRDLCRFHADDYINFLRSVTPETQQDQIR...LLKRFNV  110
SbHDA6     SEDDLLVVHSESYLNSLKSSAKVARIVEVPAVALLPNLLV  124
SbHDA7     SSPMTQTYQIPPRPHAAPADYYDATGAKTRRREAAEGYNL  132
SbHDA8     YPIELAQFHSADYVEFLHRITPDTQHLYAS...ELTRYNL   92
SbHDA9     SEADIRRFHSDDYVAFLASATGNPSVLDPR...AVKRFNV  109
SbHDA10    HASELRSFHSSEYIEELVQANATGAKKLCEG....TFLNP  121
SbHDA11    CKEDLLVVHTEAYLNSLKSSFRVSNIVEVPPVSLVPNWIV  125
SbHDA12    RDRDLCRFHADDYVAFLRSVTPETQQDQIR...ALKRFNV  109
SbHDT1     REFLIATLSDDKYPHHRTDLYLEKEFELLHSSKTRSISVI   94
SbHDT2     KKLVLGTLSVEKHPQISCDLVFDKDFELSHNSKTASVFFC   89
SbHDT3     QKLAIGTLSIDKFPQVQFDLVFDKEFELSHTSKTTSVFFS   88
SbHDT4     EKLAIGTLSIDKNPQIQCDLVFDKEFELSHTSKTTSVFLT   88
SbHDT5     KKFLIGTLSVDKYPQYTTSLVFEKEFELLHTSKTSTISAL  267
SbSRT1     THMALVELERGGFLKFVISQNVDSLHLRSGFPREKLAELH  134
SbSRT2     ASASVEAFMQPTYTCNGTQIIAGLTGALWAASRRSYAHFC  125
Consensus

SbHDA1     ESVEQTKNMLYSYFTSDTYANGHSACAAKLAAGLCADLAS  318
SbHDA2     Y..................FNKGSSESAVLAAGSVIEVAE  128
SbHDA3     G.VLKEG...E..TFYDNPPIAGLWDYCQRYAGGSLAAAR  144
SbHDA4     T..............ETTFQESLLSAGAGITLVDSVVAAS  190
SbHDA5     G..............EDCPVFDGLYSFCQTYAGASVGGAV  136
SbHDA6     Q...................QKLLYPFRKQVGGSILSAKL  145
SbHDA7     GEVWNER...ARCVTNENPVIDNLWDYCRRYAGGSLAAAR  169
SbHDA8     G..............EDCPVFDNLFEFCQIYTGGTLDAAR  118
SbHDA9     G..............EDCPVFDGLFPFCQASAGGSIGAAV  135
SbHDA10    G......................SWGAVLLAAGTTLSAVK  139
SbHDA11    H...................KKLLYPFRKQVGGSILSAKL  146
SbHDA12    G..............EDCPVFDGLYSFCQTYAGGSVGGAV  135
SbHDT1     G...................NNFS..NLEIKPDTSSKQG.  112
SbHDT2     G...................YKSPVPQFESYSGEDSSDEE  110
SbHDT3     G...................YKVEQPMEEDEMDLDSEDEE  109
SbHDT4     G...................YKVEQPTEEDEMDLDSEDEE  109
SbHDT5     G...................YKFRHCNRKYDSETSTEEGD  288
SbSRT1     G.....NSFKEICPCCKTEYLRDFEIETIGLKDTPRRCSD  169
SbSRT2     N..............FQASGNNALLYGRENPLSLRYSFRF  151
Consensus

SbHDA1     LMVSGRVRNGFALVRPPGHHAGVKQAMGFCLHNNAAVAAL  358
SbHDA2     KVAAGELSSAIALVRPPGHHAEHDEAMGFCLFNNVAVAAN  168
SbHDA3     ALGEGKA.DIAINWSGGMHHACKAAASGFCFVNDIVLAIK  183
SbHDA4     KLGPNPPLGFALVRPPG.HHAVPQGPMGFCVFGNIAVAAR  229
SbHDA5     KLNHG.H.DIAINWSGGLHHAKKCEASGFCYVNDIVLAIL  174
SbHDA6     ALERG....CAINIGGGFHHCSAEEGGGFCAYADISLCIH  181
SbHDA7     ALASGKY.KVAINWSGGMHHVCEGKAGGFCYVNDIV....  204
SbHDA8     RLNHKTC.DIAINWAGGLHHAKKCEASGFCYINDLVLGIL  157
SbHDA9     KLNRGDA.DITVNWAGGLHHAKKSEASGFCYVNDIVLAIL  174
SbHDA10    HILDGHGNLAYALVRPPGHHAQPGHADGYCFLNNAGLAVQ  179
SbHDA11    ALERG....WAINVGGGFHHCSADEGGGFCAYADISLCIQ  182
SbHDA12    KLNHG.H.DIAINWAGGLHHAKKCEASGFCYVNDIVLAIL  173
SbHDT1     ..............................LTAIQSS...  119
SbHDT2     L..............ETDHQIPRNEISAAKVPVKGGIKV.  135
SbHDT3     E..LDVP.VIKENGKADGKE.QKSQEKAVAAASKSSLVS.  144
SbHDT4     EEELNVP.VIKENGKAEGKEKQKSQEKAVSAASKSSLASR  148
SbHDT5     ESNEEVPQAIPLYPNADDDKSKESKCGVEKPAATESSKSK  328
SbSRT1     KNCGARLKDTVLDWDDALPPEEMNSAKEHCRSADLVLCLG  209
SbSRT2     LKARNNHSSAVAPKDYCETYIQFLRDKRIVPDSDPPSSKD  191
Consensus

SbHDA1     AAKRAG....AKKVLIVDWDVHHGNGTQEIFEGDKTVLYI  394
SbHDA2     YLLNERPDLGIKKILIVDWDVHHGNGTQKMFYNDPRVLFF  208
SbHDA3     DLLH.....HFRRVLYVDIDVHHGDGVETAFLDSNRV.MT  217
SbHDA4     YAQHQHG...LKRVMIIDFDVHHGNGTSDAFYVDPD...I  263
SbHDA5     ELLK.....HHERVLYVDIDIHHGDGVEEAFYTTDRV.MT  208
SbHDA6     FAFVR...LNISRVMIIDLDAHQGNGHEKDFGSDER...V  215
SbHDA7     ..........FRRVLYLDINAHHGDGVKTAFVDDSR....  230
SbHDA8     ELLK.....YHARVLYIDIDVHHGDGVEEAFYFTDRV.MT  191
SbHDA9     ELLK.....FHRRVLYVDIDVHHGDGVEEAFFTTNRV.MT  208
SbHDA10    LALDS....GRAKVAVVDIDVHYGNGTAEGFYRTDNVLTM  215
SbHDA11    FAFAR...LNISSVLIIDLDAHQGNGHEKDFANDGR...V  216
SbHDA12    ELLK.....YHQRVLYVDIDIHHGDGVEEAFYTTDRV.MT  207
SbHDT1     .TLEE...TMDAGKLQTGVGASDDDDSDEDYLDSEG..GE  153
SbHDT2     ....E...SSSSDDDDDDFTDSEMSDEDDSSDEDD.....  163
SbHDT3     ...KK...SKDDDDSDEDETDDSDSDDTDDSDEGEGLSPE  178
SbHDT4     LVGKK...SKDEDDSDEDETDDSDVDETDDSDEGEGLSPE  185
SbHDT5     VSLEE...AKVPDKHKADVGGDDNDESDED.VDSED..GE  362
SbSRT1     TSLQ.....ITPACNMPLMSIKNGGRVAIVNLQATPKDKK  244
SbSRT2     VDLLYQFIDKSKRLMVVTGAGMSTESGIPDYRSPNG..AY  229
Consensus

SbHDA1     SLHRHEDGN..FYPGTGAAHEVGVLDGQGFS..VNIPWSR  430
SbHDA2     SVHRFDYGS..FYPAEGDASHCFIGEEGGKGYNINVPWEH  246
SbHDA3     VSFHQRTKN..FFPEERGHVNHVGEGAGLYR.ALNVPFME  254
SbHDA4     FFLSTHQLG..SYPGTGKINEVGQGDGEGTT..LNLPLPG  299
SbHDA5     VSFHKFGD...YFPGTGDIRDIGHSKGKYYS..LNVPLDD  243
SbHDA6     YTLDMYNSG..IYPFDHVAKKYIDQKIELHS.........  244
SbHDA7     VSFHQFDGE..FFPRTGAAVDVGGGDNGVCPTLINVPLQV  268
SbHDA8     VSFHKYGDL..FFPGTGDIKDIGEREGKYYA..INIPLKD  227
SbHDA9     VSFHKYGD...FFPGTGHITDVGAAEGKHYA..LNVPLSD  243
SbHDA10    SLHMRHGSWGPSHPQSGSVDEIGEGKGLGYN..LNIPLPN  253
SbHDA11    YIFDMYNAG..IYPFDFTAKQYIDQKVELAS.........  245
SbHDA12    VSFHKFGD...YFPGTGDIRDVGHSKGKYYS..LNVPLDD  242
SbHDT1     TSDDEDFS.........SNEDD.EDSPKS.........AK  174
SbHDT2     VSTGDDSSD..D....SGSEEQ.TPTPKKTD......VVV  190
SbHDT3     EGDDDDSSD..EDD.TSDDEEEETPTPKKK.......PEA  208
SbHDT4     EGDDDDSSD..EDD.TSEGEEE.TPTPKK........PEA  213
SbHDT5     SGDDESSSD..EDDGESSNEDDDEDSPKS.........TK  391
SbSRT1     ASLVIHGLVDKVIAGVMSKLSLRIPPYIRTD....FVQLT  280
SbSRT2     STGFKPLTHQEFVRSIQARRRYWARSYAGWR.....RFRR  264
Consensus

SbHDA1     GGVGDNDYIFAFQTVVLPIAAEFAADITIISAGFDAARGD  470
SbHDA2     GKCGDADYIAAWDHVLLPVTKVFDPDIILVSAGFDAALGD  286
SbHDA3     .GVGDEGYCEMFKAIMKRVVDVFQPEAIVMQCGSDSLSGD  293
SbHDA4     .GAGDYAMRYAFDEVIAPSAHRFKPDIILVSAGYDAHVLD  338
SbHDA5     .GIDDESYQSLFKPIMGKVMEVFRPGAVVLQCGADSLSGD  282
SbHDA6     .GTKTEDYLEILDKALKVAESRFQPQLILYNAGTDILDGD  283
SbHDA7     .GTRDGRYHQLFAPVVDRVMAVFEPDAVVMQCGADSLAGD  307
SbHDA8     .GIDDSSFTRLFKTIIAKVIETYLPGAIVLQCGADSLARD  266
SbHDA9     .GIDDATFRDLFQCIIKKVMEVYQPDVVVLQCGADSLAGD  282
SbHDA10    .GSGDAGYEYAMNELVVPAIDKFQPQLLVLVIGQDSSAFD  292
SbHDA11    .GTETDEYLEQLDKALEVCNSRFQPQLIVYNAGTDILDGD  284
SbHDA12    .GIDDESYQSLFKPIMGKVMEVFNPGAVVLQCGADSLSGD  281
SbHDT1     .DKNRPEEKTLKT.PPEKKARMTTPGSG......TGMRTG  206
SbHDT2     .GKKRAIQAEA...PSGKKAKSEQ.SAQKTG...DKKVST  222
SbHDT3     .GKKRPAENSL.TPLSDKKAKVATPSAQKTG...GKKGAV  243
SbHDT4     .GKKRAAENALKTLLSDKKVKVATPSAQKTG...GKKGAT  249
SbHDT5     .RNNRPAETPLKT.PPGKKARITTPSMGKKSVSDDAKRSN  429
SbSRT1     LRHSVKKKCVRWTLRVTSIHGLRAPLPFLQSVKVSFPERP  320
SbSRT2     AQPNAAHYALASLERIGRVHSMVTQNVDRLHHRAGSNPLE  304
Consensus

SbHDA1     PLGGCDVTP....VGYSWMTSLLADCSNG.RLLVILEGGY  505
SbHDA2     PLGGCCITPN...GYALLLTKLLGFAQG..RIVMALEGGY  321
SbHDA3     RLGQLNLS....IAGHAQCVSYMRSFNL..PLLLLGGGGY  327
SbHDA4     PLAGLQFTTGTFYMLAFSIKQLAKELCGG.RCVFFLEGGY  377
SbHDA5     RLGCFNLS....IRGHAECVRYMRSFNV..PLLLLGGGGY  316
SbHDA6     PLGSLKVSPDGVITRDEKVFRFAKDQSI..PLLMLTSGGY  321
SbHDA7     WLASLGLS....VRGHAKCVRIVKGYGL..PLLLLGGGGY  341
SbHDA8     RLGCFNLS....IEGHAECVKFVKKFNI..PLLVTGGGGY  300
SbHDA9     RLGCFNLS....VKGHADCLRFLRSYNV..PMMVLGGGGY  316
SbHDA10    PNGRQCLTMEGYRKIGQIMRSLANRHSNG.QILVVQEGGY  331
SbHDA11    PLGRLKVSPEGVVTRDEKVFTFAKDQNI..PLLMLTSGGY  322
SbHDA12    RLGCFNLS....IKGHAECVRFMRSFNV..PLLLLGGGGY  315
SbHDT1     YVHVATPYP.........SKLVKKTPSI..VERPKQPTGY  235
SbHDT2     HSAKQSSKT........PDKSTKTPTAD..KKSPKSG.SH  251
SbHDT3     HVATPHPAK........GKTPANNDKSK..EKSPKSGGSV  273
SbHDT4     HVATPYPAKR......AGKTPANNDKSK..ERSPRSGRSV  281
SbHDT5     HVHVATPYPS........SKQVKTARSI..IDNSKQSTGY  459
SbSRT1     DMKSVVLKEQ...PFSLQRETSMNKPFFM.LLTLNFSDGC  356
SbSRT2     LHGSVYDVICLECGTSISRESFQEEVKNLNPKWAQAIDSL  344
Consensus

SbHDA1     NLQSISSSATEVVKVLLGDGPNRASFVGSPSREALKTVSQ  545
SbHDA2     NLRSIANSVCACAKVLLGDKFTFNAPEMQPFESTWSVIQA  361
SbHDA3     TVNHVAACWCYETAVAIGKEIDDNIPVHCYDTYYSGQGYK  367
SbHDA4     NLQSLSSSVADTFRAFLDETSLAAQFDDPAILFEEPTRKI  417
SbHDA5     TIRNVARCWCYETGVALGQELEDKMPVNEYYEYFGPDYTL  356
SbHDA6     MKSSARVIADSIINLSNKNLIDIDTHCIEIE.........  352
SbHDA7     TINHVASCWCYETKKIT.......................  358
SbHDA8     TKENVARCWAVETGVLLDTELPNEIPNNEYIEYFAPDYTL  340
SbHDA9     TIRNVARCWCYETAVAVGVEPDNKLPYNDYYEYFGPDYIL  356
SbHDA10    HITYSAYCLHATLEGVLDLEAPLLDDPIAYYPEDEEYTMK  371
SbHDA11    MKSSARVIADSIINLSNKNLIGLGSELG............  350
SbHDA12    TIRNVARCWCYETGVALGHELTDKMPPNEYYEYFGPDYTL  355
SbHDT1     ACNSCSRTFNSYIALKTHCKVKKHKRSPHSSEMS......  269
SbHDT2     ACKSCNKSFSSATGLESHQKAKKHEA..............  277
SbHDT3     PCKSCTKTFNSEMALQAHSKAKHGAK..............  299
SbHDT4     PCKSCSKTFNSEMALQAHSKAKHRAK..............  307
SbHDT5     ACKSCSKTFYSSVGLETHCKVKHSACK.............  486
SbSRT1     SCLSSSIGWPVDFQKQKDSFVRDRALVLKELQSAAEHETC  396
SbSRT2     EVGQPGSDKSFGMQQRPDGDVEIDEKFWEQDFEIPSCHQC  384
Consensus

SbHDA1     VLKIQQRFWPVLGPTYASLQAQQGSVSSNHITKRNELKKR  585
SbHDA2     VRNELKTCWPVLSSKLPENVSLRIRPSPSEVNASSDSESD  401
SbHDA3     LH........YPVDKQLKNENKRSYLERTMTAVLENLSHL  399
SbHDA4     KDAIEKAKGIHSL...........................  430
SbHDA5     HV........APSN..MENKNTRNQLDDIRSKLLDNLSKL  386
SbHDA6     ........................................  352
SbHDA7     ........................................  358
SbHDA8     KV........PNLN..MDNLNSKTYLSSIKVQVMESLRSI  370
SbHDA9     HI........QPKS..VENQNTTKDLENIKNMILESLSKI  386
SbHDA10    VVDMVRSCWKESVPFLKDI.....................  390
SbHDA11    ........................................  350
SbHDA12    HV........APSN..MENKNTRHQLDDIKSKLLDNLSKL  385
SbHDT1     ........................................  269
SbHDT2     ........................................  277
SbHDT3     ........................................  299
SbHDT4     ........................................  307
SbHDT5     ........................................  486
SbSRT1     GGQ......QEVLEREILPRAETSIHGIVTNVVRYDIEAE  430
SbSRT2     GGVLKPDVVMFGDNVPQERAESAKEAARTCDALLVVGSAL  424
Consensus

SbHDA1     KHSGGLGPFWWKLGSKRLLYEALFEHRRQRKIKGSGEGKA  625
SbHDA2     SEDVAELLPTVASVNVIEVAGDAISEHLSKMKLDDDNLSV  441
SbHDA3     EAAPSVQFQDPPGPGGSGSTNTEPLFYEQAPREDDDPMER  439
SbHDA4     ........................................  430
SbHDA5     RHAPSVQFQERVPDTEVPEPDEDQDDPDERHDPDSDMEVD  426
SbHDA6     ........................................  352
SbHDA7     ........................................  358
SbHDA8     QHAPGVQMQEIFISQILTKMNWILMNALTSIPKTSRFTVM  410
SbHDA9     EHVPSTQFHDRPSDPEAPE..EKEEDMDKRPAQRSRLWSG  424
SbHDA10    ........................................  390
SbHDA11    ........................................  350
SbHDA12    RHAPSVQFQERPPEAELPEQDEDKENPDERRDADSDVEMN  425
SbHDT1     ........................................  269
SbHDT2     ........................................  277
SbHDT3     ........................................  299
SbHDT4     ........................................  307
SbHDT5     ........................................  486
SbSRT1     KLAPPKDDLMNHSRSNPAKRHMEGTDCHSPLPKKLKYFLK  470
SbSRT2     MTMSAFRLARLAHEAHAPIAAVSIGETRADSILSLKINAR  464
Consensus

SbHDA1     TGFSSTLDG...............................  634
SbHDA2     KTTSSCSAAEQHLVDSVKVQNNASVVLTKKISDLSLAWRS  481
SbHDA3     LHRLCGERDERCFFRELGKKRQLANFFA............  467
SbHDA4     ........................................  430
SbHDA5     DHKAVEESARR.SIPGIKVKREFGENETKVQDGGRIASDH  465
SbHDA6     ........................................  352
SbHDA7     ........................................  358
SbHDA8     MSTMKVT...........MTTITTMAHANLLPRNRCKWKL  439
SbHDA9     GAYDSDTEDPD......NLKSESKDVTANLQMKDEPKNDL  458
SbHDA10    ........................................  390
SbHDA11    ........................................  350
SbHDA12    DAKPLDDYGRRGSIQNLRMKKESAETEAVDQDVNSAAAEH  465
SbHDT1     ........................................  269
SbHDT2     ........................................  277
SbHDT3     ........................................  299
SbHDT4     ........................................  307
SbHDT5     ........................................  486
SbSRT1     DERLNC..................................  476
SbSRT2     CGEILPRILQMGSLVVPNIS....................  484
Consensus

SbHDA1     ........................................  634
SbHDA2     DLSRTDVWYASFGSNMWRPRFLCYVQGGKAEGMSIACYGS  521
SbHDA3     ........................................  467
SbHDA4     ........................................  430
SbHDA5     .RGVEPMAEDIGSSKQ.APADASAMAVDEPSNVKNEPESS  503
SbHDA6     ........................................  352
SbHDA7     ........................................  358
SbHDA8     LLCSSAAL................................  447
SbHDA9     ........................................  458
SbHDA10    ........................................  390
SbHDA11    ........................................  350
SbHDA12    SRGTGPVADGVGSSKQTFPNDTTPMSIDEPNVLKVEQQSP  505
SbHDT1     ........................................  269
SbHDT2     ........................................  277
SbHDT3     ........................................  299
SbHDT4     ........................................  307
SbHDT5     ........................................  486
SbSRT1     ........................................  476
SbSRT2     ........................................  484
Consensus

SbHDA1     ........................................  634
SbHDA2     RDTSSPKGTMWKTVPHRLLFGRSSTPCWGTGGVAFLNPEI  561
SbHDA3     ........................................  467
SbHDA4     ........................................  430
SbHDA5     TKLQDQAAAYHKP...........................  516
SbHDA6     ........................................  352
SbHDA7     ........................................  358
SbHDA8     ........................................  447
SbHDA9     ........................................  458
SbHDA10    ........................................  390
SbHDA11    ........................................  350
SbHDA12    NKLQDQPTMHQKP...........................  518
SbHDT1     ........................................  269
SbHDT2     ........................................  277
SbHDT3     ........................................  299
SbHDT4     ........................................  307
SbHDT5     ........................................  486
SbSRT1     ........................................  476
SbSRT2     ........................................  484
Consensus

SbHDA1     ........................................  634
SbHDA2     NYNEKSYVCMYKITLEQFNDILFQENRLVLKDGKDGNVVY  601
SbHDA3     ........................................  467
SbHDA4     ........................................  430
SbHDA5     ........................................  516
SbHDA6     ........................................  352
SbHDA7     ........................................  358
SbHDA8     ........................................  447
SbHDA9     ........................................  458
SbHDA10    ........................................  390
SbHDA11    ........................................  350
SbHDA12    ........................................  518
SbHDT1     ........................................  269
SbHDT2     ........................................  277
SbHDT3     ........................................  299
SbHDT4     ........................................  307
SbHDT5     ........................................  486
SbSRT1     ........................................  476
SbSRT2     ........................................  484
Consensus

SbHDA1     ........................................  634
SbHDA2     PDSPLIGSSEIKFISTNKAIHLEPIKDSWYSNVLYLGNEG  641
SbHDA3     ........................................  467
SbHDA4     ........................................  430
SbHDA5     ........................................  516
SbHDA6     ........................................  352
SbHDA7     ........................................  358
SbHDA8     ........................................  447
SbHDA9     ........................................  458
SbHDA10    ........................................  390
SbHDA11    ........................................  350
SbHDA12    ........................................  518
SbHDT1     ........................................  269
SbHDT2     ........................................  277
SbHDT3     ........................................  299
SbHDT4     ........................................  307
SbHDT5     ........................................  486
SbSRT1     ........................................  476
SbSRT2     ........................................  484
Consensus

SbHDA1     ........................................  634
SbHDA2     ELPILTMTCPASDIERFKSGELPLAPPSETYAATLIRGLV  681
SbHDA3     ........................................  467
SbHDA4     ........................................  430
SbHDA5     ........................................  516
SbHDA6     ........................................  352
SbHDA7     ........................................  358
SbHDA8     ........................................  447
SbHDA9     ........................................  458
SbHDA10    ........................................  390
SbHDA11    ........................................  350
SbHDA12    ........................................  518
SbHDT1     ........................................  269
SbHDT2     ........................................  277
SbHDT3     ........................................  299
SbHDT4     ........................................  307
SbHDT5     ........................................  486
SbSRT1     ........................................  476
SbSRT2     ........................................  484
Consensus

SbHDA1     .....................                     634
SbHDA2     EGKQLDADGAANYINACAAKG                     702
SbHDA3     .....................                     467
SbHDA4     .....................                     430
SbHDA5     .....................                     516
SbHDA6     .....................                     352
SbHDA7     .....................                     358
SbHDA8     .....................                     447
SbHDA9     .....................                     458
SbHDA10    .....................                     390
SbHDA11    .....................                     350
SbHDA12    .....................                     518
SbHDT1     .....................                     269
SbHDT2     .....................                     277
SbHDT3     .....................                     299
SbHDT4     .....................                     307
SbHDT5     .....................                     486
SbSRT1     .....................                     476
SbSRT2     .....................                     484
Consensus
```
