# Supplementary figures and images for: Characterization of histone deacetylases and their roles in response to abiotic and PAMPs stresses in Sorghum bicolor
Source: BMC Genomics. 2022 Jan 6;23:28. doi: 10.1186/s12864-021-08229-2 (PMC8739980; doi:10.1186/s12864-021-08229-2)

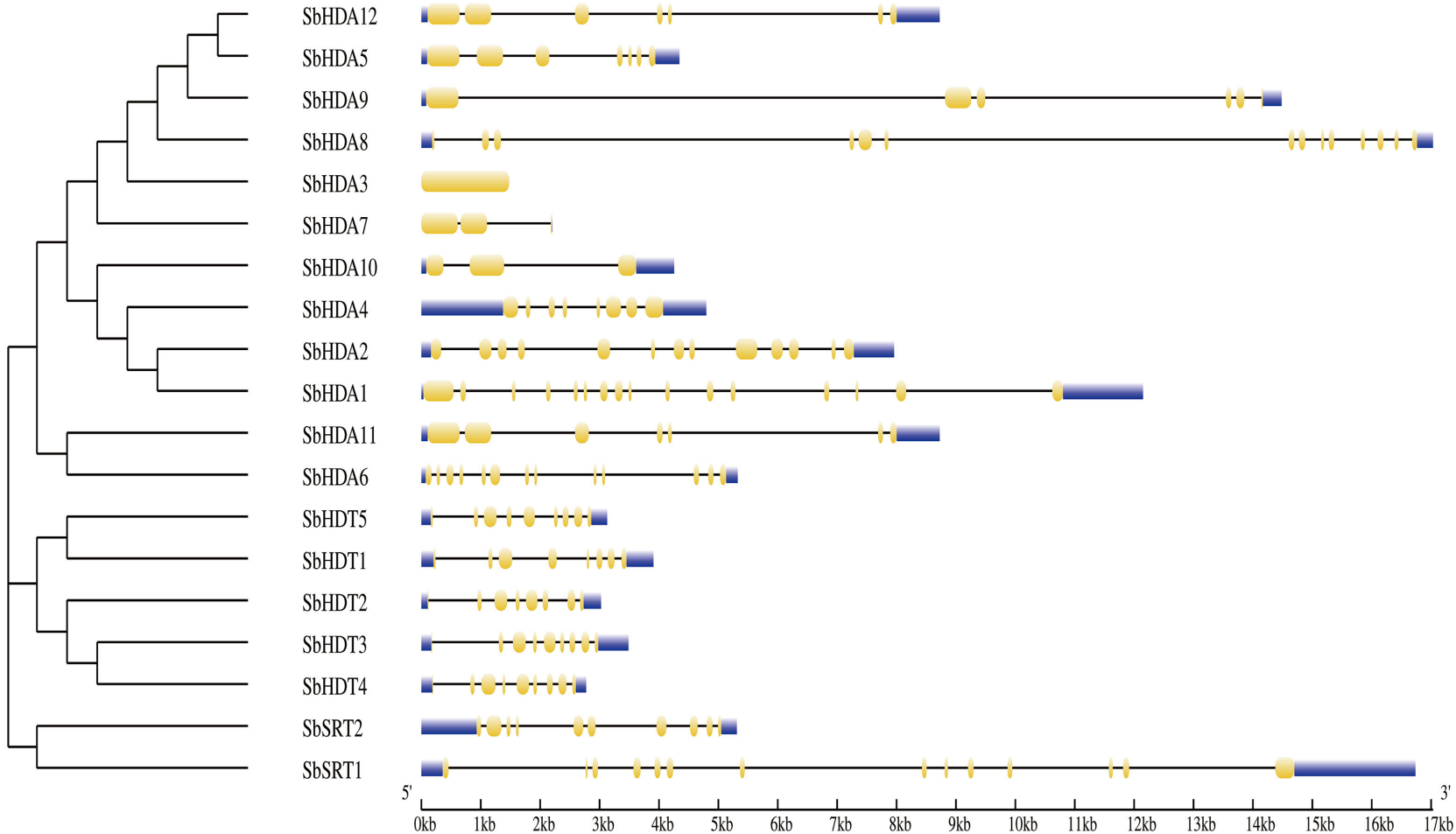

Supplement: Supplementary file 3 — Additional file 3: Figure S3. Phylogenetic relationships and gene structures of the SbHDAC family. Exons and introns were shown by filled boxes and single lines, respectively. [file 12864_2021_8229_MOESM3_ESM.pdf]

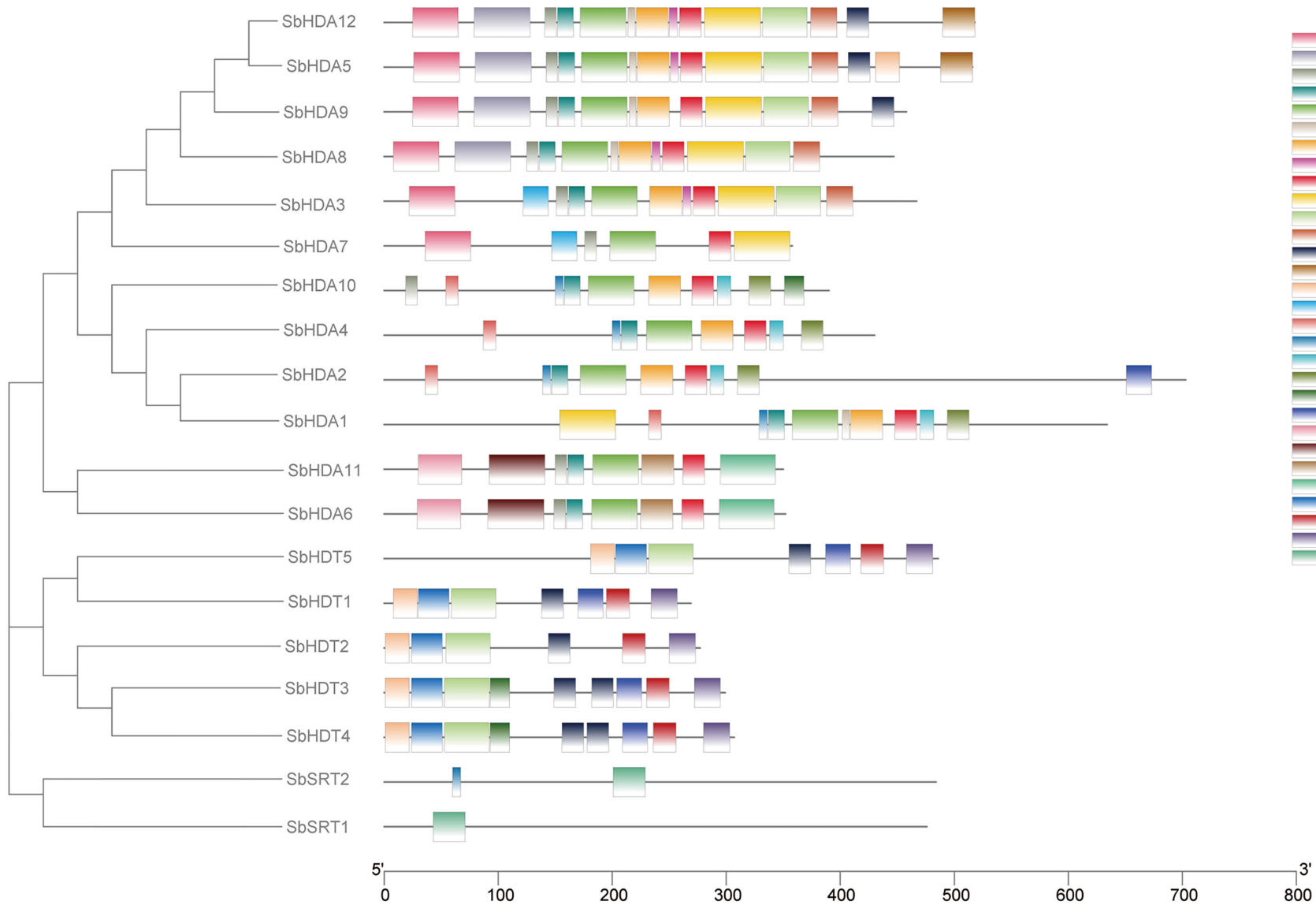

Supplement: Supplementary file 4 — Additional file 4: Figure S4. Motif analysis of SbHDAC proteins. [file 12864_2021_8229_MOESM4_ESM.pdf]

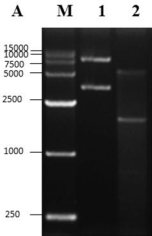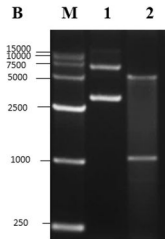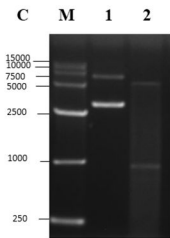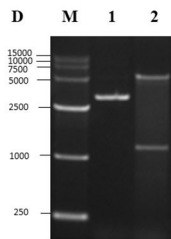

Supplement: Supplementary file 5 — Additional file 5: Figure S5. Amplification and plasmids construction of SbHDAC genes into pET28a. A-D: lane 1, recombinant plasmids. lane 2, double digestion of SbHDA1-pET28a, SbHDA3-pET28a, SbHDT3-pET28a, and SbSRT2-pET28a respectively. [file 12864_2021_8229_MOESM5_ESM.pdf]

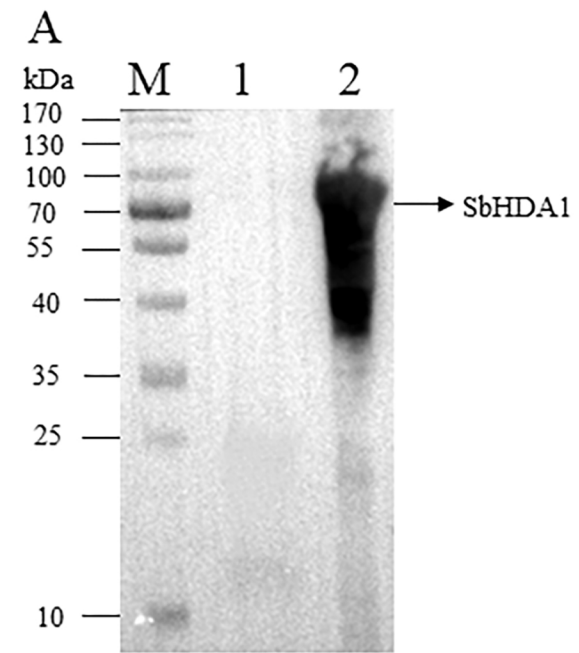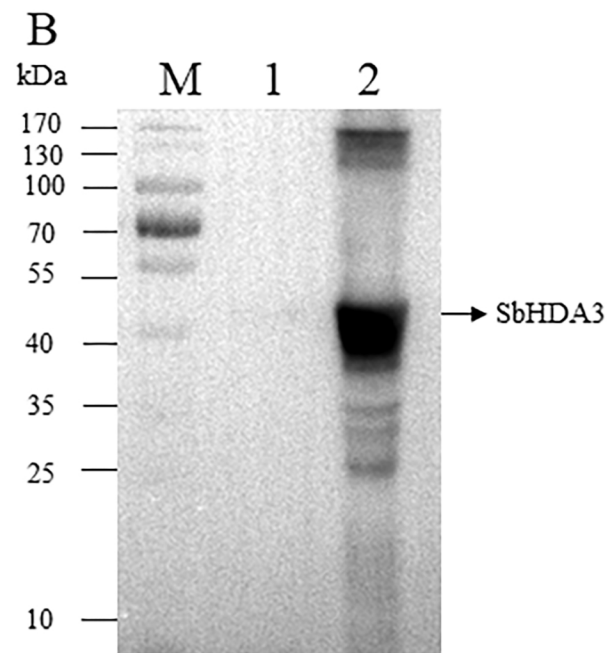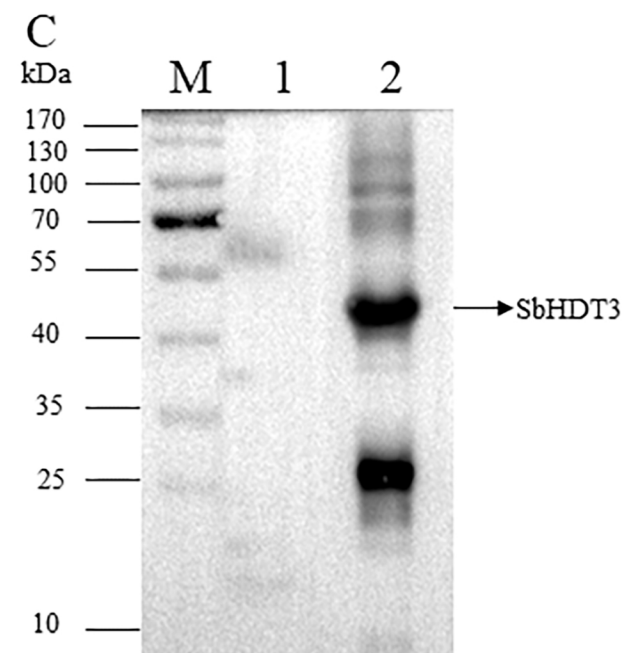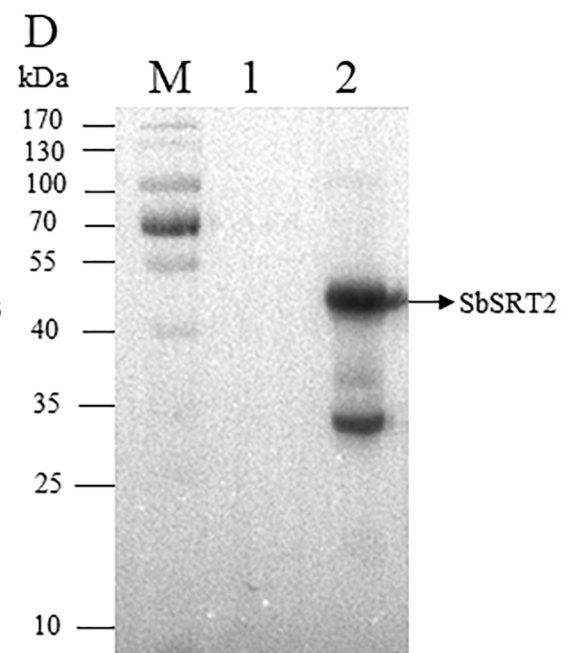

Supplement: Supplementary file 6 — Additional file 6: Figure S6. Detection of SbHDA1, SbHDA3, SbHDT3 and SbSRT2 protein by western blot. M: Protein marker; Lane1: Supernatant of empty vector; Lane 2: Supernatant of SbHDA1, SbHDA3, SbHDT3 and SbSRT2 recombinant cells. [file 12864_2021_8229_MOESM6_ESM.pdf]
