# Supplementary material for: Characterization of histone deacetylases and their roles in response to abiotic and PAMPs stresses in Sorghum bicolor
Source: BMC Genomics. 2022 Jan 6;23:28. doi: 10.1186/s12864-021-08229-2 (PMC8739980; doi:10.1186/s12864-021-08229-2)
Supplement: Supplementary file 7 — Additional file 7: Table S1. Primer sequences. [file 12864_2021_8229_MOESM7_ESM.docx]

**Table S1. Primer sequences**

| Gene Name | Forward primer sequence（5‘-3’） | Reverse primer sequence（5‘-3’） |
| --- | --- | --- |
| *SbHDA1* | GAGCAGACAAAGAACATGCTTT | CCATCAAACTAGCAAGATCAGC |
| *SbHDA2* | TGTACAGCCAGTAGGTAGTAGT | GTATGTCAGACAAGAACGATGC |
| *SbHDA3* | GACTTTCTACGACAACCCTCC | TCATCACACGGTTGGAGTC |
| *SbHDA4* | TTCTCTAGATGATGTTGCACGA | TCATCTGAAGCTCTACGCATAG |
| *SbHDA5* | GACCGAAGTGAATCTGCAATAC | GTATACATGGGTCAGACTACCG |
| *SbHDA6* | AGTCGTACTTGAACAGCCTTAA | TATCAGCATATGCACAAAAGCC |
| *SbHDA7* | TCGTTCCACCAGTTCGAC | GTTGATCAGAGTAGGGCACAC |
| *SbHDA8* | CTCATCGTCTCTGTATGACACA | GAGTTATCCGGTGCAAGAATTC |
| *SbHDA9* | CTTAACCATCTCAGGCGAAATG | GATCGACGGCTCGTAGAAATAG |
| *SbHDA10* | GAAAGTTGTCGACATGGTAAGG | TGCAATATTGTTTGTGACCAGG |
| *SbHDA11* | TCAAAAGCAGCTTCAGAGTTTC | GAACAATGGTGAAATCCTCCAC |
| *SbHDA12* | CTACAGTTTCTGCCAGACCTAC | CGCTGGTGATACTTTAGTAGCT |
| *SbHDT1* | GCCTAAGCAACCTACTGGATAT | CACCTTGCAATGAGTTTTCAGA |
| *SbHDT2* | CGAGTGAGAATGTCATTGTGTC | TTGGAATTTGGTGGTCAGTTTC |
| *SbHDT3* | CACCAAGACTTTCAACAGTGAG | TTCCAGACTCACTTAAACCTCC |
| *SbHDT4* | GCACACATCAAAAACTACCAGT | GCTTTTCTTTCCAACAAGCCTA |
| *SbHDT5* | GGCTGATGGCAAAAAGTTTCTA | CATCACCCTCTTCAGTACTTGT |
| *SbSRT1* | CAGTTTCGTCAGAGATAGAGCT | TCATACCTGACGACGTTTGTTA |
| *SbSRT2* | ACGGATAGTTCCTGATTCAGAC | AATAGAGCGAACAAACTCCTGA |
| *SbEIF4a* | AGGATTGGCACCAGAAGGGT | CACATCAAGCCCCTTGCAGA |
