# Supplementary material for: Characterization of histone deacetylases and their roles in response to abiotic and PAMPs stresses in Sorghum bicolor
Source: BMC Genomics. 2022 Jan 6;23:28. doi: 10.1186/s12864-021-08229-2 (PMC8739980; doi:10.1186/s12864-021-08229-2)
Supplement: Supplementary file 8 — Additional file 8: Table S2. Cis-acting element analysis of the SbHDAC gene family. [file 12864_2021_8229_MOESM8_ESM.docx]

**Table S2. Cis-acting element analysis of the *SbHDAC* gene family**

| Cis-acting element | Function | | Quantity |  |
| --- | --- | --- | --- | --- |
| ARE  TATA- box  CAAT-box  DRE core  MYB  MYC  STRE  LTR  MBS  TCA-element  Sp1  LAMP-element  P-box  MBS  DRE core  G-box  ABRE  RY-element | | essential for the anaerobic inductio  core promoter element  cis-elements in promoter and enhancer regions  dehydration, low temp, salt sress responsive  drought-related  drought-related  unknow  dehydration, low temp, salt sress responsive  dehydration, low temp, salt sress responsive  involved in salicylic acid responsiveness  light responsive element  part of a light responsive element  gibberellin-responsive element  MYB binding site involved in drought-inducibility  MeJA-responsiveness  involved in light responsiveness  involved in the abscisic acid responsiveness  involved in seed-specific regulation | 30  266  320  10  100  28  36  5  11  15  13  2  10  10  10  20  25  1 |  |
